# One strategy does not fit all: determinants of urban adaptation in mammals

**DOI:** 10.1111/ele.13199

**Published:** 2018-12-20

**Authors:** Luca Santini, Manuela González‐Suárez, Danilo Russo, Alejandro Gonzalez‐Voyer, Achaz von Hardenberg, Leonardo Ancillotto

**Affiliations:** ^1^ Department of Environmental Science Institute of Water and Wetland Research Radboud University Nijmegen The Netherlands; ^2^ Ecology and Evolutionary Biology School of Biological Sciences University of Reading Whiteknights Reading RG6 6AS UK; ^3^ Wildlife Research Unit Dipartimento di Agraria Università degli Studi di Napoli Federico II via Università 100 I‐80055 Portici, Napoli Italy; ^4^ Instituto de Ecología Departamento de Ecología Evolutiva Universidad Nacional Autónoma de México Ciudad México 04510 México; ^5^ Conservation Biology Research Group Department of Biological Sciences University of Chester Parkgate Road Chester CH1 4BJ UK

**Keywords:** Brain mass, diet diversity, life history, mammals, synurbic, urban ecology

## Abstract

Urbanisation exposes wildlife to new challenging conditions and environmental pressures. Some mammalian species have adapted to these novel environments, but it remains unclear which characteristics allow them to persist. To address this question, we identified 190 mammals regularly recorded in urban settlements worldwide, and used phylogenetic path analysis to test hypotheses regarding which behavioural, ecological and life history traits favour adaptation to urban environments for different mammalian groups. Our results show that all urban mammals produce larger litters; whereas other traits such as body size, behavioural plasticity and diet diversity were important for some but not all taxonomic groups. This variation highlights the idiosyncrasies of the urban adaptation process and likely reflects the diversity of ecological niches and roles mammals can play. Our study contributes towards a better understanding of mammal association to humans, which will ultimately allow the design of wildlife‐friendly urban environments and contribute to mitigate human‐wildlife conflicts.

## Introduction

In the last 50 years, the human population living in urban areas has increased from approximately 1 to up to 4 billion people (Seto *et al*. 2010). Even though the rate of urbanisation in developed countries is slowing down, it is accelerating at dramatic rates in developing areas of Africa, Asia and Latin America (Seto *et al*. 2010). This has prompted a dramatic expansion of urban areas globally, encroaching upon 0.3% of the total land area in 2000 (Angel *et al*. 2005), a trend that is projected to exacerbate in the near future, with cities expected to expand by 2.5 times in area (1.1% of the total land area) by 2030 (Angel *et al*. 2005; Seto *et al*. 2012), making urbanisation a worldwide issue for conservation.

Although urban encroachment jeopardises natural habitats by replacing or fragmenting them, it can nonetheless represent an opportunity to exploit novel environmental conditions and resources for some species. Wildlife in urban areas is exposed to novel environmental pressures including high vehicular and pedestrian traffic, large‐scale occurrence of impervious surfaces, chemical, acoustic, and light pollution (Grimm *et al*. 2008). Urban areas generally present higher temperatures than their surroundings (i.e. ‘heat island effect’; Oke 1982), thus potentially prolonging favourable climatic conditions. Increased waste production as well as the presence of introduced species such as ornamental plants, or direct feeding by people, may increase food availability (Chamberlain *et al*. 2009), while buildings and infrastructure may provide new shelters (Lowry *et al*. 2013).

Wildlife can either avoid or adapt by different degrees to urban areas (a process called synurbisation). This translates into an overall impoverishment in the diversity of animal communities along urbanisation gradients (Blair 1996; Clergeau *et al*. 1998; Marzluff 2001; Hamer 2011), delineating a picture of a few ‘winners’, well adapted to urban environments, versus many ‘losers’ whose populations decline and eventually go locally extinct (Grimm *et al*. 2008). A number of processes underlie the biodiversity loss due to urbanisation, mostly related to species’ lack of adaptations for exploiting the novel resources and avoiding risks of the urban environment (Croci *et al*. 2008). In birds, urbanisation acts as a filter to avian communities, with cities hosting a relatively limited number of species characterised not by a single particular trait, but by a combination of winning traits (Croci *et al*. 2008). Phenotypic plasticity, behavioural flexibility, dispersal abilities and niche generalism seem to have an important role for many bird taxa to cope with challenges posed by human modified habitats (Bonier *et al*. 2007; Kark *et al*. 2007; Møller 2008, 2009, 2013; Evans *et al*. 2011; Maklakov *et al*. 2011; Fristoe *et al*. 2017).

Mammals represent an interesting model to test hypotheses regarding the traits that favour adaptation to urban environments. Given their high diversity in body structure, size, life‐history and ecology, selective pressures in urban habitats may have contrasting effects on different mammalian groups, making the global picture potentially more complex than in birds. Several mammalian species are known to occur in urban ecosystems worldwide (termed synurbic species, henceforth urban species), either occasionally (urban visitors) or permanently (urban dwellers), with many exhibiting higher densities in urban environments than in natural habitats (Baker *et al*. 2003). Terrestrial mammals display a range of locomotion modalities (aerial, semiaquatic, fossorial, terrestrial and arboreal), and body size varies considerably across orders, ranging from 2.5 g Etruscan shrews *Suncus etruscus* found in settlements across the Mediterranean basin (Gippoliti & Amori 2006), to 90 kg leopards *Panthera pardus* roaming in the Indian suburbs (Athreya *et al*. 2016). Urban species may also show extreme variation in ranging behaviour, with species rarely moving distances > 100 m such as the house mouse *Mus domesticus* (Mikesic & Drickamer 1992), to others travelling up to 45 km each night from roost to foraging sites, such as the Mexican free‐tailed bat *Tadarida brasiliensis* (Best & Geluso 2003). Similarly, mammals show a great variety of diet specialisations, as well as reproductive strategies along the fast‐slow continuum in life history (Bielby *et al*. 2007), and different cognitive abilities (Willemet 2013). Given this variability, a key question is whether particular traits affect success in exploiting novel conditions, such as those offered by urban ecosystems, across all mammals and whether idiosyncrasies exist across mammalian taxa in the trait combinations that influence urbanisation tolerance.

In many cases, the presence of mammals in urban areas brings conflicts with people – including zoonotic risks, damage to structures or goods, traffic accidents, direct attacks to humans or domestic animals, or negative consequences of digging, garbage raiding or defecating (Bateman & Fleming 2012). In a global scenario of urban expansion (Angel *et al*. 2005; Seto *et al*. 2012), conflicts between humans and wild mammals are likely to exacerbate. Identifying the biological traits favouring synurbisation is therefore pivotal to inform current management, as well as to generate predictions for the future.

To tackle this challenge, here we analyse the direct and indirect effects of behavioural, ecological and life history traits on mammalian ability to exploit urban environments using phylogenetic path analysis. We focused on a number of biological traits as proxies of evolutionary, demographic and behavioural adaptability to conditions found in urban areas in mammals. Specifically, we focus on proxies of ranging and dispersal abilities, behavioural and cognitive plasticity, diet generalism and reproductive rates to test specific, non‐mutually exclusive, causal hypotheses of the relationship between these traits and synurbisation in mammals.

## Methods

### Data sources and species categorisation

We collected species‐average values of body mass, wing morphology (bats), brain mass, diet, weaning age, and litter size from publicly available databases and the literature. Data on body mass and diet were obtained from the EltonTrait database (Wilman *et al*. 2014). Brain mass data were obtained from multiple sources (Mace *et al*. 1981; Jeschke & Strayer 2006; Pitnick *et al*. 2006; Isler & Van Schaik 2009; Weisbecker & Goswami 2010; Barton & Capellini 2011; Boddy *et al*. 2012; DeCasien *et al*. 2017; Stankowich & Romero 2017; Razafindratsima *et al*. 2018). Data on litter size and weaning age were obtained from the PanTHERIA (Jones *et al*. 2009), Anage (Tacutu *et al*. 2013) and Amniote databases (Myhrvold *et al*. 2015). We estimated diet diversity by calculating a Shannon Index on the proportions of 10 food item categories, as reported in EltonTraits (Wilman *et al*. 2014). Data on bat wing morphology were retrieved from Norberg & Rayner (1987) and other published references (see Table S1).

Since species characterised by a different ecology may show distinct traits that prove successful in urban environments, and an uneven species richness per group might lead to an overestimate of the effect of traits possessed by the most speciose groups, we built and tested separate causal models for the different taxonomic orders. This approach also contributed to reduce possible taxonomic biases in data collection. Furthermore, to avoid comparing species from different regions, for each mammalian order, we restricted groups of non‐urban species to those found in the same biogeographic realms (as defined in Holt *et al*. 2013) of the urban species in the dataset.

Urban species have been defined based on comparisons between urban and non‐urban populations using different approaches in the literature (Fischer *et al*. 2015). In terms of demography, urban taxa are defined as those whose population densities are, in urban ecosystems, greater than in natural ones (Møller *et al*. 2012). A fitness‐based criterion, instead, assumes increasing reproductive success in urban areas from the so‐called ‘avoiders’, through ‘adapters’, and, finally ‘exploiters’ (Møller 2009). For mammals, the necessary information to apply such definitions is scarce and unevenly distributed across orders, so we adopted a spatial/functional definition, classifying species according to the available evidence of the use that different species make of urban habitats. First, we reviewed the literature using scientific search engines (Web of Science, Google Scholar), entering the following keywords and their combinations: wildlife*, urbanisation* OR urbanization*, urban mammals*, name of taxon* (at order level). We excluded all references reporting occasional species in urban areas (single records), species found in artificial structures (e.g. buildings) when these were actually isolated within extensive natural habitats, as well as records referring only to genera. The species retrieved were classified as follows: a) urban ‘dwellers’ – species that exploit urban areas to fulfil all their needs (breeding, foraging) including those that do so in green areas embedded in an urban matrix; b) urban ‘visitors’ – species that occur in urban areas but exploit resources from a nearby natural matrix and to do so regularly leave the urban area; or those that make sporadic incursions into urban environments. Species that met both criteria (i.e. in different studies) were assigned to both groups. Species that were unambiguously classified as urban visitors were excluded from the analysis of urban dwellers, while those unambiguously classified as urban dwellers were excluded from the analysis of urban visitors. The full list of urban mammals included in this study is available as Table S2. Such a discretisation along what is actually a continuum of adaptations to urban environments is inevitable due to the lack of detailed knowledge on mammals’ response to urbanisation. Therefore, we do not aim to estimate the contribution of each trait to the degree of adaptation, but rather the extent to which traits increase the probability of different uses (visitors or dwellers) of such environments, a necessary first‐step in our understanding of the process.

Species can use different habitats within urban contexts, varying from suburbs to city centres, or from small gardens to urban parks. Yet, different urban habitats are difficult to categorise objectively because they are rather extremes of a gradient. We account for this problem by running separate analyses per taxonomic order, as habitat use among different species is largely consistent within the same taxonomic order; for example, urban bats mostly roost in buildings (Russo & Ancillotto 2015), carnivores generally den in parks but forage outside (Bateman *et al*. 2012), ungulates generally visit suburbs at night (Conover 1995), and insectivores only persist in urban parks (Braaker *et al*. 2014; Vergnes *et al*. 2013).

**Figure 1:**
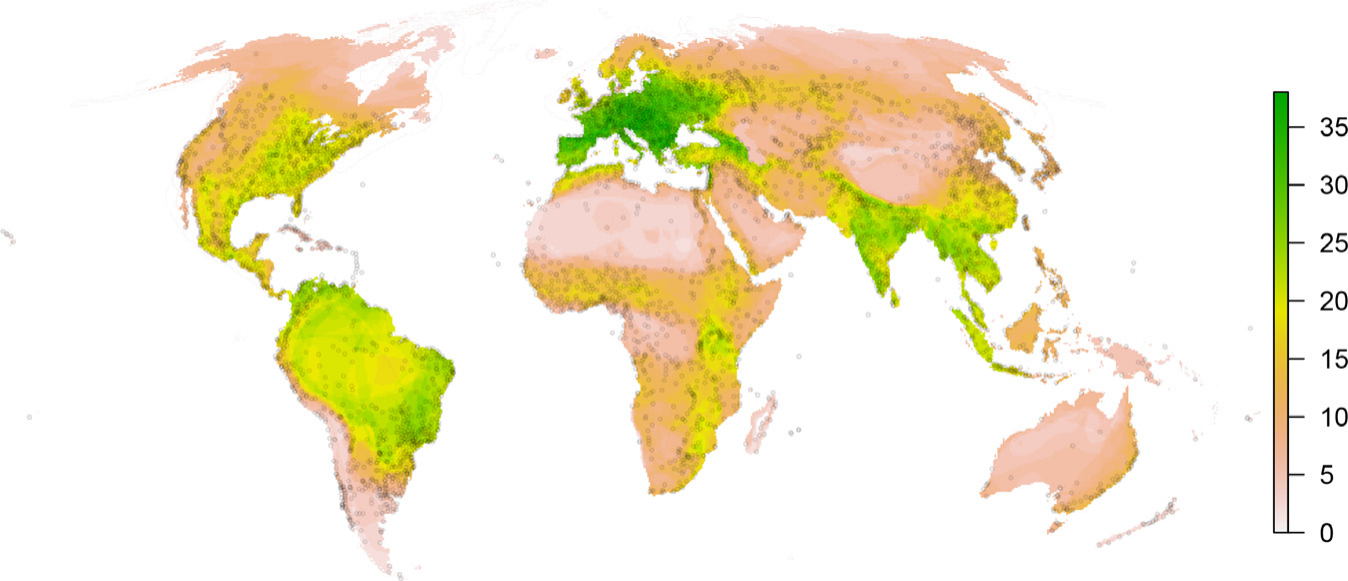
Species richness map of urban mammals. Circles represent urban settlements with > 10 000 people.

To assess any possible geographic bias in the data collection, we produced a species richness map of urban mammals (Fig. 1), using the IUCN range polygons for all urban species in our dataset (IUCN 2017). We then overlaid urban settlements worldwide with a population > 10 000 (Kelso & Patterson 2012). We used Spearman's rank correlation to measure the agreement between richness and urban density at increasing resolutions spanning from 100 km to 500 km. We varied the resolution to consider a number of factors. First, previous authors suggested using a resolution of 2° (~ 220 km at the equator; Hurlbert & Jetz 2007) to account for the spatial uncertainty of such coarse geographic range maps. Second, we were interested in geographic regions characterised by high urban densities rather than specific locations. Finally, focusing on coarse resolutions allowed us to account for recent range shifts and different times of urban expansion.

### Analyses

As a first data exploration step, we pooled urban visitor and dwellers and ran contingency table chi‐squared tests with analysis of adjusted residuals, to test whether the global mammalian species richness was proportionally represented in urban environments (1) across all orders, and (2) within each order at the family level. The adjusted residuals quantify the contribution of the contingency table cells to the significance of the overall test; values > 2 indicate a significant difference between observed and expected frequencies. When considering families, we completed two analyses: testing only families comprising at least one urban species, and a more conservative approach testing all families within an order, restricting the sample to orders comprising > 10 urban taxa. Significance level was set at α = 0.05.

To test how traits influence the ability of mammals to live in urban environments, we used phylogenetic path analysis (von Hardenberg & Gonzalez‐Voyer 2013). This approach allows comparing causal hypotheses of the relationship among traits disentangling direct from indirect effects, while correcting for the non‐independence of trait data due to common ancestry. This approach deals with multicollinearity better than multivariate linear models because the variance in the response is partitioned among fewer predictors (Gonzalez‐Voyer & von Hardenberg 2014). We excluded species with incomplete trait information, and only retained datasets of taxonomic orders that included at least 20 species. For each taxonomic order and urban condition (urban visitors or dwellers), we tested the hypotheses listed in Table 1. We used a two‐step approach to define a set of hypotheses (depicted by directed acyclic graphs) to minimise the number of models to test (Gonzalez‐Voyer *et al*. 2016). First, for each taxonomic order, we identified a taxon‐specific model representing the relationships between body size, brain size, life‐history traits and diet (hereafter termed ‘trait‐only model’). In these models, we only considered significant paths, and we ensured that all conditional independencies (i.e. non‐significant relationships between non‐linked variables) were met (Gonzalez‐Voyer & von Hardenberg 2014). To define the trait‐only model for each group, we tested specific directional relationships based on a priori knowledge and expectations derived from published articles (Table 1). We considered body mass to possibly drive changes in all other traits (Peters 1983). Specifically, body mass can determine brain mass (Martin 1981), weaning age and litter size (Bielby *et al*. 2007). Cognitive abilities have often been considered to be linked with habitat generalism, whereas the link with diet diversity is not entirely clear (Edmunds *et al*. 2016; Navarrete *et al*. 2016); yet, foraging in different habitats can lead to an increase in diet diversity. Selective pressures on increased cognitive abilities can arise from foraging in a variety of habitats, or vice versa, increased cognitive abilities could lead to the exploration of a wider range of habitats. Here, we considered brain mass as a potential predictor of diet diversity. In turn, we considered brain mass to potentially influence reproductive traits, as suggested by previous phylogenetic path analyses on mammals (Gonzalez‐Voyer *et al*. 2016). If a relationship existed between litter size and weaning age, we assumed litter size to be the causal parent of weaning age. Finally, in bats, we assumed wing aspect ratio (i.e. the ratio between wing span and wing area; Norberg & Rayner 1987) to be potentially related to brain mass (Safi *et al*. 2005; Ratcliffe *et al*. 2006). Once we determined the trait‐only model that best described the relationships between the aforementioned traits for each group, we tested additional paths linking biological trait variables with the observed propensity to exploit urban ecosystems (as urban visitors or dwellers), generating a total of 32 models per group representing our working hypotheses and their combinations (Table 1). All tests of conditional independencies were based on phylogenetic generalised least squares models for continuous responses (Martins & Hansen 1997), and phylogenetic generalised linear model with Binomial family for binary responses (where 1 = urban visitor or dweller; 0 otherwise) (Ho & Ané 2014). To check the validity of causal relationships depicted in the path analysis models, we calculated the Fisher's C statistics and ran the d‐sep test (Gonzalez‐Voyer & von Hardenberg 2014). P‐values below an alpha value of 0.05 lead us to reject proposed independences and models. We estimated path coefficients using a model averaging approach weighting causal links by CICc weight (ω) of supported models (ΔCICc > 2) (conditional model averaging *sensu* von Hardenberg & Gonzalez‐Voyer 2013). Phylogenetic path analysis models were built and tested in R 3.3.0. (R Core Team 2018) using the development version of the ‘phylopath’ package (van der Bijl 2018) that allows including binary response variables (available at https://github.com/Ax3man/phylopath). Phylogenetic relationships were based on the updated mammalian supertree from Fritz *et al*. (2009).

**Table 1:** Hypotheses on traits pre‐adapting species to urban environments

	Hypothesis	Predictions	Variable	Taxa	Rationale
1	Null	Nothing influences ability of species to exploit urban areas	–	E, R, B, C, U, P	Factors other than the biological traits considered (including random chance) actually allow mammals to live in cities
2	Body mass	Small urban dwellers and large urban visitors	Body mass	E, R, B, C, U, P	Small body masses may allow species to hide/nest/roost more easily in wall cracks, underground, small green urban spots, bushes, canopies, etc
					Large body masses, on the other hand, are associated to larger home ranges and higher dispersal abilities (Kelt & Van Vuren 2001; Santini *et al*. 2013)
3	Diet breadth	Higher diet diversity in urban species	Shannon Index on diet	E, R, C, U, P	Diet diversity makes species more adaptable allowing them to exploit a wider range of resources, therefore making them able to colonise a wide range of habitats (Slatyer *et al*. 2013)
4	Reproductive timing	Slower and faster reproductive rates in urban species	Weaning age	E, R, B, C, U, P	Weaning age is a proxy of reproductive timing (frequency of reproductive events; Bielby *et al*. 2007). Species with slow reproductive timing are generally characterised by low mortality rates (Schaffer 1974; Charlesworth 1980; Reznick *et al*. 1990; Stearns 2000). On the one hand low weaning age may provide faster adaptive responses, increased spread rate and capacity to cope with unpredictable environment (Santini *et al*. 2016). On the other hand, high weaning age is generally associated to longer parental care that might be necessary to learn how to avoid certain threats (e.g. traffic), the development of a large brain (Gonzalez‐Voyer *et al*. 2016) and to dispersal abilities (Whitmee & Orme 2012). Therefore, both strategies can potentially advantage urban visitors and dwellers
5	Reproductive output	High reproductive output in urban dwellers and low in urban visitors	Litter size	E, R, B, C, U, P	Litter size is a proxy of reproductive output (investement; Bielby *et al*. 2007). Species producing large litters generally invest less in each newborn, therefore litter size can represent the balance between number and quality of offspring produced (Schaffer 1974; Charlesworth 1980; Reznick *et al*. 1990; Stearns 2000). Large litters increase species ability to spread and colonise new environments (Whitmee & Orme 2012; Santini *et al*. 2016), to cope with unpredictable environments characterised by high mortality rates (e.g. traffic, predation by domestic animals, human persecution)
6	Behavioural flexibility	Higher encephalization in urban species	Brain mass	E, R, B, C, U, P	A large brain for a given body mass is expected to provide adaptive benefits. The cognitive buffer hypothesis states that enhanced encephalization (large brains for a given body mass) provides adaptive benefits such as behavioural flexibility to cope with new conditions. Several papers have shown that birds living in urban environments are characterised by large brains (Maklakov *et al*. 2011; Fristoe *et al*. 2017). Others have argued that enhanced encephalisation in terrestrial vertebrates (amphibians, reptiles, birds and mammals) improves their ability to colonise and successfully establish into novel environments (Sol *et al*. 2002, 2008; Amiel *et al*. 2011). Only evidence for a small number of species is available for mammals (i.e. Snell‐Rood & Wick 2013)
7	Enhanced flying ability	High aspect ratio in urban species	Aspect ratio	B	Aspect ratio (wing span/wing area) describes wing morphology of bats, i.e. higher values indicate longer, narrower wings, positively correlates with ranging abilities and flight speed, and being associated to species that fly in open spaces or edge habitats (Jung & Kalko 2011). Urban areas are typically open habitats, thus potentially favouring species with higher aspect ratios, both for visitors and dwellers

Note

E = Eulipotyphla, R = Rodents, B = Bats, C = Carnivores, U = Ungulates, P = Primates.

## Results

### Global pattern of urban species

We found a high richness of urban species in southern and central Europe, and secondarily in the Southern part of Asia (Indo–Chinese region), Eastern Australia, Eastern Africa, Western North America, and Northern Latin America (Fig. 1). The Spearman's rank correlation coefficient between urban species richness and the density of urban settlements ranged from 0.47 to 0.84 (depending on the resolution; Fig. S1), indicating a moderate to very good agreement between the recorded urban species and highly urbanised regions worldwide.

### Species occurring in urban areas

**Figure 2:**
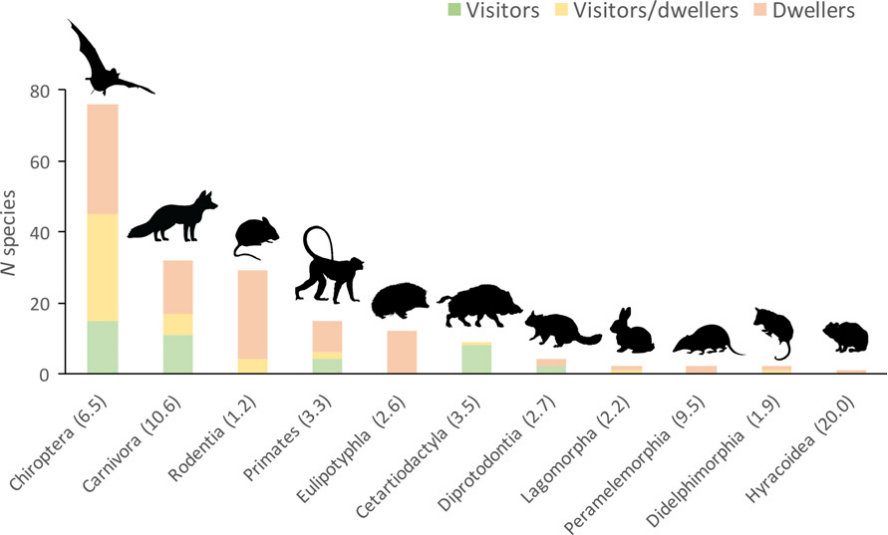
Numbers of mammal species per order found in urban environments. Numbers in parentheses indicate percentage of urban species within the order. The visitors/dwellers category reflects species that due to ambiguous evidence from the literature were included as visitors and as dwellers in the analyses.

We classified 190 species as urban, of which 39 were urban visitors, 105 urban dwellers, and 46 were assigned to both categories (Table S2, Fig. 2). Most of our data come from urban checklists (~ 70%; Table S2), thus limiting the risk of taxonomic biases in published articles. The most frequent orders represented in urban mammalian communities were bats (Chiroptera; 78 species), carnivores (Carnivora; 36 species), rodents (Rodentia; 28 species) and primates (Primates; 15 species); other taxa include insectivores (Eulipotyphla), ungulates (Cetartiodactyls), lagomorphs (Lagomorpha), hyraxes (Hyracoidaea) and marsupials (Didelphimorphia and Diprotodontia), with variable numbers (range: 1–12 taxa per group).

The richness of urban mammals per order is not representative of the overall species richness observed in the mammalian orders (χ^2^ = 130.68, d.f. = 4, *P* < 0.001), with Chiroptera and Carnivora being significantly over‐represented in urban habitats (adjusted cell residuals > 2 for both groups). Within these two orders, only bats showed a family‐biased abundance across urban taxa, with Molossidae featuring significantly more urban species (*n* = 29; adjusted cell residuals > 2) than other bat families, both with the conservative (χ^2^ = 82.24, d.f. = 4, *P* < 0.001) and relaxed (χ^2^ = 67.21, d.f. = 2, *P *< 0.001) approaches. This family represents over one‐third (*n* = 29) of bat species found urban areas worldwide.

### Urban‐related traits

The final datasets for urban visitors included ungulates (*n* = 68), carnivores (*n *= 63) and bats (*n* = 47); whereas the final datasets for urban dwellers included rodents (*n* = 202), insectivores (*n* = 24), bats (*n* = 52), primates (*n* = 132) and carnivores (*n* = 92).

**Figure 3:**
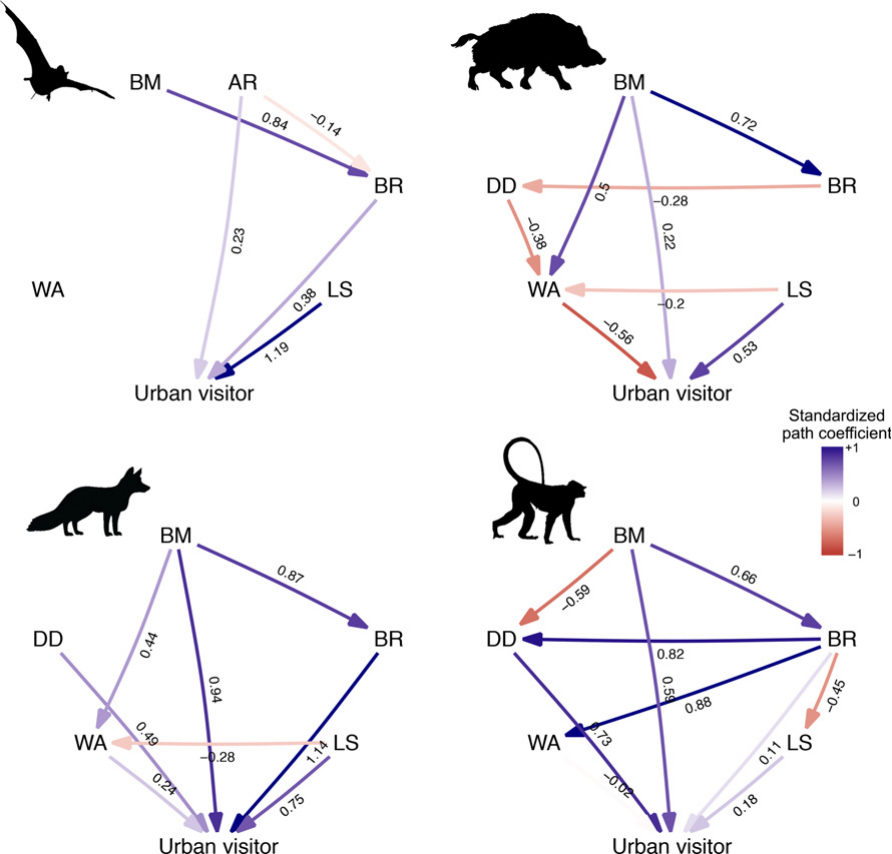
Average models for urban visitors. Values represent standardised average coefficients. BM = Body mass; AR = Aspect Ratio; DD = Diet diversity (not modelled in bats); BR = Brain mass; WA = Weaning age; LS = Litter size. Silhouettes indicate mammalian orders as in Fig. 1.

**Figure 4:**
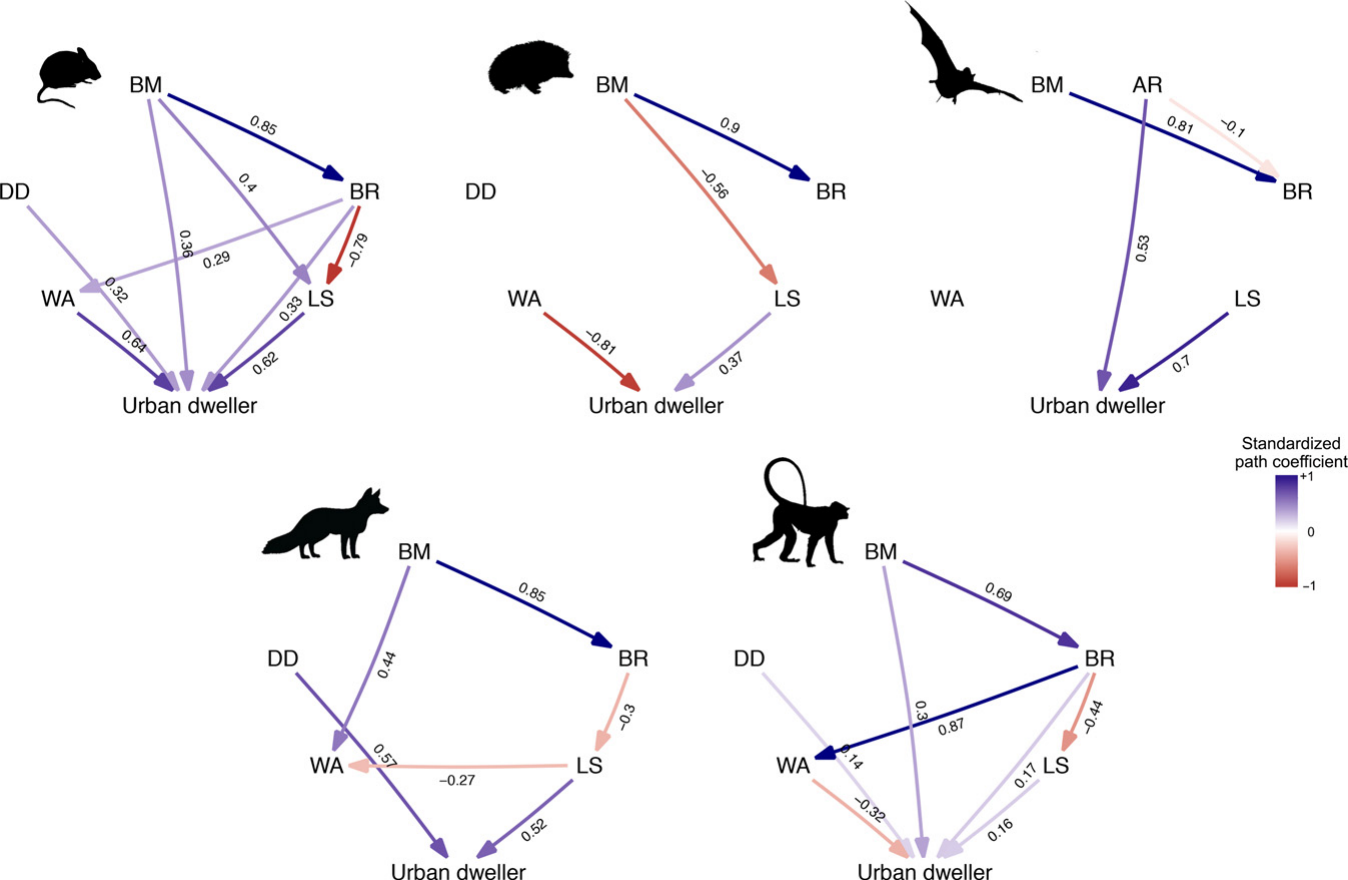
Average models for urban dwellers. Values represent standardised average coefficients. BM = Body mass; AR = Aspect Ratio; DD = Diet diversity (not modelled in bats); BR = Brain mass; WA = Weaning age; LS = Litter size. Silhouettes indicate mammalian orders as in Fig. 1.

Model selection offered support to our original hypotheses (Table 1), but effects were context‐ and group‐dependent, with different traits found to influence propensity to use urban areas for species classified as urban visitors or dwellers, and differences among orders (Table 2). Nonetheless, larger litter sizes stand out as consistently associated with adaptation to urban environments across all mammalian orders tested (Table 2, Figs 3 and 4). As predicted, we also found brain mass to be larger in carnivores, bats and primates among urban visitors, and in primates and rodents among urban dwellers (Table 2, Figs 3 and 4), suggesting an advantage associated with behavioural flexibility. Furthermore, as predicted, we found that carnivores, ungulates and primates occasionally visiting urban areas were larger than non‐urban species; yet, contrary to our prediction, primates and rodents among urban dwellers were also larger (Table 2, Figs 3 and 4). Diet diversity was high in urban dwellers and visitors for both carnivores and primates and in rodent urban dwellers (Table 2, Figs 3 and 4). Reproductive timing (weaning age) was important as predicted, with later weaning ages for carnivore visitors and rodent dwellers, and earlier for ungulate visitors, primates and insectivore dwellers (Table 2, Figs 3 and 4). Finally, a high wing aspect ratio was, as predicted, an important factor for bat visitors and dwellers (Table 2, Figs 3 and 4). Overall, all hypotheses were supported for some groups with consistent effects, except for the effect of weaning age that varied across groups.

**Table 2:** Model selection summary table only including models for which conditional independencies are met and ΔCICc < 2

Urban	Group	Model	q	C	p	CICc	ΔCICc	ω	BM	AR	DD	BR	WA	LS
Urban visitor	Chiroptera	LS	9	16.53	0.87	39.39	0	0.29						1.21 (0.45)*
	Chiroptera	BR + LS	10	14.68	0.88	40.79	1.4	0.14				0.38 (0.55)		1.27 (0.51)*
	Chiroptera	AR + LS	10	14.81	0.87	40.92	1.52	0.13		0.23 (0.53)				1.08 (0.43)*
	Carnivora	BM + DD + LS	12	21.94	0.23	51.14	0	0.22	1.59 (0.6)*		0.48 (0.38)			0.9 (0.46).
	Carnivora	DD + BR + LS	12	23.08	0.19	52.28	1.14	0.13			0.52 (0.42)	1.71 (0.62)*		1 (0.49)*
	Carnivora	BM + BR + LS	12	23.44	0.17	52.64	1.51	0.11	−0.46 (0.38)			1.03 (0.47)*		0.39 (0.16)*
	Carnivora	BR + WA + LS	12	23.78	0.16	52.98	1.85	0.09				0.47 (0.2)*	0.24 (0.16)	0.41 (0.16)*
	Cetartyodactyla	WA + LS	13	11.46	0.78	44.2	0	0.29					−0.53 (0.3).	0.57 (0.35)
	Cetartyodactyla	BM + WA + LS	14	9.8	0.78	45.73	1.52	0.14	0.22 (0.28)				−0.62 (0.33).	0.45 (0.31)
	Primates	BM + DD + LS	14	15.03	0.38	46.68	0	0.25	0.64 (0.36).		0.76 (0.36)*			0.16 (0.16)
	Primates	BM + DD + WA + LS	15	14.01	0.3	48.22	1.54	0.12	0.61 (0.3)*		0.71 (0.27)*		−0.02 (0.13)	0.22 (0.11).
	Primates	BM + DD + BR + LS	15	14.44	0.27	48.65	1.97	0.09	0.41 (0.46)		0.65 (0.26)*	0.11 (0.46)		0.19 (0.13)
Urban dweller	Rodentia	WA + LS	12	16.24	0.58	41.9	0	0.12					0.72 (0.36)*	0.64 (0.32)*
	Rodentia	BR + WA + LS	13	14.66	0.55	42.6	0.71	0.08				0.17 (0.36)	0.55 (0.4)	0.64 (0.31)*
	Rodentia	BM + WA + LS	13	14.71	0.55	42.65	0.75	0.08	0.23 (0.33)				0.57 (0.39)	0.62 (0.31)*
	Rodentia	BM + DD + WA + LS	14	12.48	0.57	42.73	0.83	0.08	0.36 (0.35)		0.34 (0.31)		0.52 (0.38)	0.6 (0.31)*
	Rodentia	DD + WA + LS	13	14.87	0.53	42.8	0.91	0.07			0.32 (0.29)		0.89 (0.44)*	0.76 (0.36)*
	Rodentia	DD + BR + WA + LS	14	13.21	0.51	43.46	1.56	0.05			0.28 (0.3)	0.31 (0.37)	0.51 (0.4)	0.71 (0.31)*
	Rodentia	LS	11	20.22	0.44	43.61	1.72	0.05						0.34 (0.21)
	Rodentia	BM + DD + LS	13	15.87	0.46	43.81	1.91	0.04	0.62 (0.31)*		0.33 (0.31)			0.56 (0.3).
	Rodentia	DD + BR + LS	13	15.93	0.46	43.87	1.98	0.04			0.31 (0.31)	0.59 (0.33).		0.6 (0.31)*
	Rodentia	BR + LS	12	18.23	0.44	43.88	1.98	0.04				0.42 (0.32)		0.69 (0.31)*
	Eulipotyphla	Trait‐only	8	32.01	0.19	57.61	0	0.28						
	Eulipotyphla	WA	9	27.41	0.29	58.27	0.66	0.2					−0.81 (0.63)	
	Eulipotyphla	LS	9	28.19	0.25	59.04	1.43	0.14						0.37 (0.54)
	Chiroptera	AR + LS	10	18.34	0.69	43.7	0	0.43		0.53 (0.37)				0.7 (0.32)*
	Carnivora	DD + LS	12	15.64	0.62	43.59	0	0.36			0.57 (0.29)*			0.52 (0.27).
	Primates	BM + BR + WA + LS	13	18.38	0.3	47.47	0	0.14	0.49 (0.28).			0.15 (0.3)	−0.5 (0.19)*	0.21 (0.1)*
	Primates	DD + BR + WA	12	21.05	0.28	47.67	0.21	0.13			0.12 (0.08)	0.19 (0.15)	−0.28 (0.15).	
	Primates	DD + BR + WA + LS	13	18.65	0.29	47.73	0.27	0.13			0.11 (0.1)	0.16 (0.21)	−0.13 (0.13)	0.11 (0.11)
	Primates	BR + DD + WA + LS	13	19.08	0.26	48.16	0.69	0.1	0.36 (0.2).		0.18 (0.11).		−0.3 (0.17).	0.2 (0.11).
	Primates	BM + DD + WA	12	21.81	0.24	48.44	0.97	0.09	0.2 (0.12)		0.14 (0.08).		−0.33 (0.15)*	
	Primates	DD + BR + LS	12	21.98	0.23	48.61	1.14	0.08			0.14 (0.08).	0.03 (0.1)		0.1 (0.08)
	Primates	BM + DD + BR + WA	13	19.79	0.23	48.87	1.41	0.07	−0.06 (0.26)		0.15 (0.11)	0.33 (0.35)	−0.37 (0.19)*	

Notes

q = number of parameters estimated in the path model; C = Fisher's C statistic; p = *P*‐value of the Fisher's C statistic obtained through the d‐sep test; CICc = C statistic Information Criterion with correction for small sample sizes; ΔCICc = difference between the CIC of the best model and subsequent models; ω = CICc weights that represent the probability of each path model given the data and the set of models being compared; Standardised path coefficients (SE): Body mass (BM), Aspect ratio (AR), Diet diversity (DD), Brain mass (BR), Weaning age (WA) and Litter size (LS). Confidence interval not overlapping with zero: * = 95%; . = 90%. Only hypothesised direct links between biological traits and propensity to urbanisation are presented for each model as direct links between biological traits (‘trait only model’) do not vary among different causal models.

## Discussion

We found that a high diversity of mammals is regularly recorded in urban settlements worldwide, comprising ca. 3.4% of global mammal species and representing more than 50% of extant mammalian orders. Our results support different hypothesised effects of ecological and life history traits on the likelihood of mammalian species to behave as urban dwellers or visitors across different orders. In most cases, more than one trait appears to be involved in the adaptation success with only litter size found to be important across all taxonomic groups. Our results highlight the filtering effect on traits that predispose species to persist in urban environments, rather than an actual selection process. This does not rule out the existence of evolutionary changes in species living in urban areas, as found by previous studies, although evidence for this is available for a limited number of species (e.g. Snell‐Rood & Wick 2013; Tomassini *et al*. 2014).

Generally, reproduction‐related traits were important determinants of success in urban environments across all mammalian orders. In particular, high reproductive output seems to have been a winning pre‐adaptation in all taxonomic groups, likely facilitating the exploitation of urban environments, and suggesting that the high mortality rates in urbanised environments represent a major selective pressure for mammals. Extrinsic mortality in such environments can be due to factors such as roadkill (Bateman & Fleming 2012), conflict with humans, or predation by domestic cats, dogs, or opportunistic birds such as corvids (Rodewald *et al*. 2011; Ancillotto *et al*. 2013). Higher reproductive outputs may thus represent a fundamental trait to counterbalance increased mortality; for example, most urban‐dwelling primates belong to species that often give birth to twins (e.g. tamarins and marmosets; Price 1992), while most urban rodents (from genera *Rattus* and *Mus*) typically produce multiple large litters in relatively short time intervals (Brooks & Jackson 1973). Even in the case of slowly reproducing mammals such as bats, whose reproductive output is strongly affected by the evolution of active flight and thus usually limited to one pup per litter produced each year (Crichton & Krutzsch 2000), larger litter sizes are observed in the case of urban species, which typically produce litters of 2 (*Nyctalus* spp., *Pipistrellus* spp.) and up to 4 (*Lasiurus* spp.) pups (Kurta & Kunz 1987). These results align with what is predicted by theory, with high reproductive output favoured in unpredictable environments (Schaffer 1974). Weaning age has a positive effect on rodents and carnivore visitors, potentially due to the increasing learning opportunities for juveniles to acquire skills that could be valuable to exploit urban areas (Gittleman 1994). The same trait had a negative effect in other groups (ungulates, and primate and insectivore dwellers), possibly reflecting an advantage of fast adaptive responses (Carlisle 1982).

Brain mass, a trait found to correlate with behavioural flexibility across different taxa (Lefebvre *et al*. 2004; Sol *et al*. 2008; Fristoe *et al*. 2017), appears to be associated to urbanisation in mammals with positive effects mostly in urban visitors (carnivores, bats and primates), and less frequently in urban dwellers (primates and rodents). Urban species may actually face increased frequency of unpredictable conditions, continuously facing the challenges from both natural and modified habitats by regularly moving between the two. As found for urban birds (Maklakov *et al*. 2011; Fristoe *et al*. 2017), larger brain mass in mammals may determine the ability to cope with such high unpredictability (Sol *et al*. 2008), particularly in groups such as bats and carnivores, whose cognitive abilities are often complex (Safi & Dechmann 2005; Bailey *et al*. 2013). Previous studies found support for the hypothesis that rural environments select for increased cranial volumes in small mammals, and a filtering effect of urban environments towards larger brain sizes possibly associated with increased behavioural plasticity (Snell‐Rood & Wick 2013).

Close proximity to humans provides novel food types and foraging opportunities to commensal wildlife, such as those offered by garbage dumps (Yom‐Tov 2003). In addition, the food provided by humans, introduced taxa, and domestic animals may present a supplemental food resource for those species able to exploit it (e.g. Prange *et al*. 2003; Athreya *et al*. 2013). We found that high diet diversity is an important predictor of mammalian adaptation to urban environments in carnivores, primates and rodents. Typical urban species belonging to these mammalian orders exhibit a broad trophic niche, and include some of the most successful urban exploiters such as raccoons (*Procyon lotor*), red foxes (*Vulpes vulpes*), golden jackals (*Canis aureus*; Bateman & Fleming 2012), as well as macaques (Macaca sp. Jaman & Huffman 2013; Maibeche *et al*. 2015), and murids (Brooks & Jackson 1973).

Body mass plays an important role in determining the likelihood of urban adaptation in mammals, both directly and indirectly, but with variable directions in different groups. Urban visiting carnivores, ungulates and primates were larger than non‐urban species, probably due to the higher dispersal and ranging abilities of larger species in these groups (Kelt & Van Vuren 2001; Santini *et al*. 2013). In contrast to what we originally expected, rodent and primate dwellers were also positively related to large body sizes. Potential advantages of larger sizes include predation deterrence (e.g. by domestic animals) (Childs 1996), increased coping abilities with unpredictable food shortages, and better ranging abilities to access patchily distributed resources (Kelt & Van Vuren 2001; Santini *et al*. 2013). Interestingly, our approach highlighted an apparently contrasting effect: urban species tend to exhibit large body sizes but also large litters and fast development times. Indeed, body mass is normally inversely correlated with these two reproductive strategies in mammals (Bielby *et al*. 2007). As biological traits are linked in our model, this indicates that adaptation to urban environments is favoured when body size is large and litter sizes are larger and development times faster than expected for a given size.

Bats species with high aspect ratio values, i.e. with long narrow wings, are those most often featuring urban habits (Jung & Kalko 2011). The most common urban bats are in fact molossids and pipistrelles, two groups of aerial hawkers that hunt in open spaces and edge habitats, respectively (Russo & Ancillotto 2015). Similar patterns have been described for birds, which are more often urban when adapted to fly in open spaces (Croci *et al*. 2008). This suggests a convergent selective pressure for birds and bats in urban environments.

For many of the groups model selection showed some degree of uncertainty with several supported competing models. Yet, except for the insectivores that were characterised by a small sample size, the set of supported models (i.e. within 2 CICc units from the best model) did not include the trait‐only model (no causal path between traits and the likelihood of being a city visitor or dweller), suggesting that including direct paths to urban adaptation substantially increases the fit of the models. The existence of competing models may be explained by several statistical and biological factors. First, the number of synurbic species in mammals is extremely low compared to the total number of mammal species, so the binomial models used to test the relationship and the conditional independence between traits and urban condition were zero‐inflated, leading to low statistical power and higher uncertainty. Although we partly controlled for this by limiting the comparison to only species within the same taxonomic groups and geographic realms, the samples were still biased towards non‐urban species reflecting the reality that most mammals are not visiting or living in urban spaces. In some groups, there are few urban species, which may limit the generalizability of our inferences even if these are statistically supported (e.g. primate visitors, Table S3). Second, not all species that could potentially exploit urban environments are likely to be currently classified as urban, because being present in urban environments is also a matter of opportunity in space and time. For instance, some species only use particular urban areas within their range (e.g. the red fox: Larivière & Pasitschniak‐Arts 1996), whereas others may not be in direct contact with urban environments (e.g. tropical forest species). Different conditions, such as the amount of green areas in urban contexts, may also influence a species’ ability to use these habitats (Baker *et al*. 2003; Angold *et al*. 2006; Bateman & Fleming 2012). Therefore, many of the species classified as non‐urban might in fact be potential urban visitors or dwellers and share the same traits of those classified as urban, consequently diluting the detected effects. Nevertheless, the support of different hypotheses is also likely to reflect the diversity of strategies for mammalian adaptation to urban environments among the orders we examined.

In this study, we focused on traits for which clear hypotheses and expectations could be made based on previous knowledge. We clearly cannot test traits for which data are available for a limited number of species or that are too variable within single species. For example, activity pattern can certainly play a role in the use of urban areas; however, contrary to birds, activity pattern is extremely flexible in mammals, and except for a few very specialised taxa (e.g. bats being mostly nocturnal), any described pattern is representative of a given population rather than the species as a whole (Halle & Stenseth 2000; Curtis & Rasmussen 2006). A recent meta‐analysis shows that species in disturbed habitats shift their activity to less‐disturbed time windows (Gaynor *et al*. 2018). Furthermore, as discussed above, we believe that considering the urban habitat as a whole is an acceptable simplification in the case of mammals given our current knowledge, yet a diversity of urban habitats and conditions exist, which affects species ability to persist (Sol *et al*. 2014). Exploring the effect of urbanisation gradients on mammal species may be an interesting future avenue of research.

Urbanisation acts as a filter on mammal communities by selecting species characterised by a number of winning traits that vary across mammalian orders. On the contrary, urban birds appear to have more consistent traits, often being generalists in terms of niche position (i.e. typical niche relative to all other species; Evans *et al*. 2011) and possessing higher cognitive skills (Maklakov *et al*. 2011; Fristoe *et al*. 2017). For mammals, we found that producing more offspring is a common strategy, but the role of other traits seem to be taxon‐dependent, likely due to the overwhelming array of morphological, physiological, ecological and behavioural adaptations that arose from the radiation process of this vertebrate class (Meredith *et al*. 2011). Differences between birds and mammals may also reflect the different levels of human persecution they suffer. Mammals are commonly regarded as pests (Baker & Harris 2007), and directly or indirectly persecuted by different means depending on the group (Vuorisalo *et al*. 2001). This may have selected, or filtered, species employing a wider array of strategies than birds.

In this study we highlight the contribution of different traits to species’ ability to persist in urban contexts, and the diversity of winning strategies in mammals. Yet, we still lack a good understanding of single species responses in terms of fitness, use of habitat and resources, and evolutionary implications of living in urban areas. Future studies are needed to better explore these aspects. As urbanisation proceeds, an increasing number of mammal species are expected to adapt to urbanised environments, while others may be lost from the mammalian assemblages in urban areas. Urban areas will, therefore, be progressively important as novel settings for mammal research, conservation and management (Grimm *et al*. 2008). Our results provide a first step towards a better understanding of the traits that influence mammal association to humans. This knowledge will be key for 21st century conservationists to be able to design wildlife‐friendly urban environments and mitigate conflicts with humans.

## Authorship

LA and LS conceived the original idea. LS, MGS, DR, AGV, AvH and LA designed the research. LA, LS and MGS collected the data. LS performed the analysis. LS and LA wrote the manuscript with significant contributions from all coauthors.

## Data Availability

All the datasets are accessible from Figshare: https://doi.org/10.6084/m9.figshare.7380638.v1.
