# Supplementary material for: One strategy does not fit all: determinants of urban adaptation in mammals
Source: Ecol Lett. 2018 Dec 20;22(2):365–76. doi: 10.1111/ele.13199 (PMC7379640; doi:10.1111/ele.13199)
Supplement: Supplementary file 1 [file ELE-22-365-s001.docx]

**Supporting Information**

**One strategy doesn’t fit all: determinants of urban adaptation in mammals**

Luca Santini ^1*^, Manuela González-Suárez ^2^, Danilo Russo ^3^, Alejandro Gonzalez-Voyer ^4^, Achaz von Hardenberg ^5^, Leonardo Ancillotto ^3^

^1^ Department of Environmental Science, Institute of Water and Wetland Research, Radboud University, Nijmegen, The Netherlands. [luca.santini.eco@gmail.com](mailto:luca.santini.eco@gmail.com)

^2^ Ecology and Evolutionary Biology, School of Biological Sciences, University of Reading, Whiteknights, Reading RG6 6AS, United Kingdom.

^3^ Wildlife Research Unit, Dipartimento di Agraria, Università degli Studi di Napoli Federico II, via Università 100, I-80055 Portici, Napoli, Italy.

^4^ Instituto de Ecología, Universidad Nacional Autónoma de México, Cd México, Mexico.

^5^ Conservation Biology Research Group, Department of Biological Sciences, University of Chester, Parkgate Road, Chester CH1 4BJ, United Kingdom.

* Corresponding author: [luca.santini.eco@gmail.com](mailto:luca.santini.eco@gmail.com)

**Table S1.** Values of Aspect Ratio (wing span / wing area) for 81 bat species, and relative reference.

| **Species** | **Aspect Ratio** | **Ref N** |
| --- | --- | --- |
| *Artibeus jamaicensis* | 6.4 | 1 |
| *Artibeus lituratus* | 6.1 | 1 |
| *Asellia tridens* | 6.9 | 2 |
| *Balionycteris maculata* | 8.1 | 1 |
| *Cardioderma cor* | 5.2 | 3 |
| *Carollia perspicillata* | 6.1 | 1 |
| *Cynopterus brachyotis* | 7.7 | 1 |
| *Cynopterus horsfieldii* | 7.9 | 1 |
| *Cynopterus sphinx* | 6.7 | 1 |
| *Desmodus rotundus* | 6.7 | 1 |
| *Eidolon helvum* | 6.9 | 1 |
| *Eonycteris spelaea* | 8.6 | 1 |
| *Epomophorus gambianus* | 5.9 | 1 |
| *Epomophorus wahlbergi* | 6.1 | 1 |
| *Eptesicus fuscus* | 6.4 | 1 |
| *Eptesicus serotinus* | 6.5 | 1 |
| *Eumops perotis* | 9.5 | 1 |
| *Glossophaga soricina* | 6.4 | 1 |
| *Hipposideros ater* | 5.5 | 4 |
| *Hipposideros caffer* | 6.3 | 1 |
| *Hipposideros speoris* | 6.5 | 1 |
| *Lasiurus borealis* | 6.7 | 1 |
| *Lavia frons* | 5.4 | 1 |
| *Leptonycteris curasoae* | 5.9 | 5 |
| *Macroderma gigas* | 6.1 | 3 |
| *Macroglossus minimus* | 6.5 | 1 |
| *Megaderma lyra* | 6.2 | 1 |
| *Megaderma spasma* | 5 | 3 |
| *Micropteropus pusillus* | 8.7 | 1 |
| *Mimon crenulatum* | 8.3 | 1 |
| *Molossus molossus* | 8.7 | 1 |
| *Molossus rufus* | 11 | 1 |
| *Mops condylurus* | 9.1 | 1 |
| *Myotis albescens* | 6.9 | 1 |
| *Myotis daubentonii* | 6.3 | 1 |
| *Myotis lucifugus* | 6 | 1 |
| *Myotis myotis* | 6.3 | 1 |
| *Myotis nattereri* | 6.4 | 1 |
| *Myotis nigricans* | 6.5 | 1 |
| *Myotis tricolor* | 6.2 | 1 |
| *Myotis velifer* | 6.2 | 1 |
| *Neoromicia nanus* | 7.3 | 1 |
| *Noctilio albiventris* | 7.8 | 1 |
| *Noctilio leporinus* | 9 | 1 |
| *Nyctalus noctula* | 7.4 | 1 |
| *Nycteris grandis* | 5.2 | 1 |
| *Nycteris macrotis* | 5.2 | 1 |
| *Nycteris thebaica* | 5.5 | 1 |
| *Nyctimene robinsoni* | 5.6 | 1 |
| *Phyllostomus hastatus* | 7.6 | 1 |
| *Pipistrellus ceylonicus* | 7.9 | 1 |
| *Pipistrellus kuhlii* | 6.3 | 1 |
| *Pipistrellus subflavus* | 6.2 | 1 |
| *Pipistrellus tenuis* | 6.5 | 6 |
| *Plecotus auritus* | 5.7 | 1 |
| *Pteronotus parnellii* | 8.1 | 7 |
| *Pteropus scapulatus* | 7.3 | 8 |
| *Pteropus tonganus* | 7.1 | 1 |
| *Pteropus vampyrus* | 8.4 | 1 |
| *Rhinolophus ferrumequinum* | 6.1 | 1 |
| *Rhinolophus hipposideros* | 5.7 | 1 |
| *Rhinolophus landeri* | 6.1 | 1 |
| *Rhinolophus megaphyllus* | 6.1 | 1 |
| *Rhinopoma hardwickii* | 6.9 | 1 |
| *Rhinopoma microphyllum* | 8 | 1 |
| *Rhynchonycteris naso* | 6.5 | 9 |
| *Rousettus aegyptiacus* | 5.9 | 1 |
| *Rousettus amplexicaudatus* | 6.3 | 1 |
| *Saccopteryx bilineata* | 6.1 | 1 |
| *Scotophilus heathii* | 7.5 | 1 |
| *Scotophilus kuhlii* | 7.3 | 10 |
| *Scotorepens sanborni* | 8 | 11 |
| *Sturnira lilium* | 6.5 | 1 |
| *Syconycteris australis* | 6.2 | 1 |
| *Tadarida aegyptiaca* | 9.7 | 1 |
| *Taphozous longimanus* | 9.8 | 1 |
| *Taphozous melanopogon* | 10 | 1 |
| *Tylonycteris pachypus* | 8.3 | 12 |
| *Uroderma bilobatum* | 6.3 | 1 |
| *Vespertilio murinus* | 7 | 1 |

**Table S2.** Classification of mammals (n=182) according to their degree of synurbization (dweller, visitor or both), with relative references.

| **Order** | **Family** | **Species** | **Urban** | **Ref N** |
| --- | --- | --- | --- | --- |
| Carnivora | Canidae | *Canis aureus* | visitor | 13 |
| Carnivora | Canidae | *Canis latrans* | visitor/dweller | 14, 156 |
| Carnivora | Canidae | *Canis lupus* | visitor | 15 |
| Carnivora | Canidae | *Nyctereutes procyonoides* | visitor | 16 |
| Carnivora | Canidae | *Urocyon cinereoargenteus* | dweller | 17 |
| Carnivora | Canidae | *Vulpes vulpes* | dweller | 18, 157, 169 |
| Carnivora | Felidae | *Felis catus* | dweller | 19 |
| Carnivora | Felidae | *Panthera pardus* | visitor/dweller | 20 |
| Carnivora | Felidae | *Puma concolor* | visitor | 21 |
| Carnivora | Herpestidae | *Cynictis penicillata* | dweller | 22 |
| Carnivora | Herpestidae | *Galerella sanguinea* | dweller | 23 |
| Carnivora | Herpestidae | *Herpestes auropunctatus* | visitor/dweller | 24 |
| Carnivora | Herpestidae | *Herpestes javanicus* | dweller | 25 |
| Carnivora | Hyaenidae | *Crocuta crocuta* | visitor/dweller | 26, 158 |
| Carnivora | Hyaenidae | *Hyaena hyaena* | visitor | 27 |
| Carnivora | Mephitidae | *Mephitis mephitis* | dweller | 28 |
| Carnivora | Mephitidae | *Spilogale gracilis* | dweller | 28 |
| Carnivora | Mephitidae | *Spilogale putorius* | dweller | 29 |
| Carnivora | Mustelidae | *Lontra canadensis* | visitor/dweller | 30 |
| Carnivora | Mustelidae | *Lutra lutra* | visitor/dweller | 31 |
| Carnivora | Mustelidae | *Lutrogale perspicillata* | dweller | 32 |
| Carnivora | Mustelidae | *Martes foina* | dweller | 34, 159 |
| Carnivora | Mustelidae | *Meles meles* | dweller | 33 |
| Carnivora | Mustelidae | *Mustela nivalis* | dweller | 35 |
| Carnivora | Otariidae | *Zalophus californianus* | visitor | 36 |
| Carnivora | Phocidae | *Phoca vitulina* | visitor | 37 |
| Carnivora | Procyonidae | *Nasua narica* | visitor | 38 |
| Carnivora | Procyonidae | *Procyon lotor* | dweller | 39 |
| Carnivora | Ursidae | *Melursus ursinus* | visitor | 40 |
| Carnivora | Ursidae | *Ursus americanus* | visitor/dweller | 42, 160 |
| Carnivora | Ursidae | *Ursus arctos* | visitor | 15 |
| Carnivora | Viverridae | *Genetta genetta* | dweller | 15 |
| Carnivora | Viverridae | *Genetta tigrina* | dweller | 43, 161 |
| Carnivora | Viverridae | *Paguma larvata* | dweller | 44, 162 |
| Carnivora | Viverridae | *Paradoxurus ermaphroditus* | dweller | 45, 162 |
| Carnivora | Viverridae | *Viverricula indica* | dweller | 46, 162 |
| Cetartiodactyla | Cervidae | *Axis axis* | visitor | 46 |
| Cetartiodactyla | Cervidae | *Capreolus capreolus* | visitor | 47 |
| Cetartiodactyla | Cervidae | *Cervus elaphus* | visitor | 48 |
| Cetartiodactyla | Cervidae | *Cervus nippon* | dweller | 44 |
| Cetartiodactyla | Cervidae | *Dama dama* | visitor | 49 |
| Cetartiodactyla | Cervidae | *Muntiacus reevesi* | visitor/dweller | 23 |
| Cetartiodactyla | Cervidae | *Odocoileus virginianus* | visitor | 50 |
| Cetartiodactyla | Suidae | *Sus scrofa* | visitor | 51 |
| Cetartiodactyla | Tayassuidae | *Pecari tajacu* | visitor | 52 |
| Cetartiodactyla | Tayassuidae | *Tayassu pecari* | visitor | 53 |
| Chiroptera | Emballonuridae | *Saccolaimus saccolaimus* | dweller | 54 |
| Chiroptera | Emballonuridae | *Taphozous melanopogon* | dweller | 54 |
| Chiroptera | Molossidae | *Chaerephon chapini* | visitor | 55 |
| Chiroptera | Molossidae | *Chaerephon leucogaster* | dweller | 56 |
| Chiroptera | Molossidae | *Chaerephon plicatus* | visitor | 57 |
| Chiroptera | Molossidae | *Cynomops abrasus* | visitor/dweller | 58 |
| Chiroptera | Molossidae | *Cynomops greenhalli* | visitor/dweller | 59 |
| Chiroptera | Molossidae | *Cynomops planirostris* | visitor/dweller | 59 |
| Chiroptera | Molossidae | *Eumops auripendulus* | dweller | 60 |
| Chiroptera | Molossidae | *Eumops bonariensis* | dweller | 60 |
| Chiroptera | Molossidae | *Eumops glaucinus* | visitor/dweller | 58 |
| Chiroptera | Molossidae | *Eumops hansae* | visitor/dweller | 58 |
| Chiroptera | Molossidae | *Eumops maurus* | visitor/dweller | 58 |
| Chiroptera | Molossidae | *Eumops perotis* | visitor/dweller | 58 |
| Chiroptera | Molossidae | *Molossus currentium* | visitor/dweller | 59 |
| Chiroptera | Molossidae | *Molossus molossus* | visitor/dweller | 61 |
| Chiroptera | Molossidae | *Molossus rufus* | visitor/dweller | 62 |
| Chiroptera | Molossidae | *Mops condylurus* | visitor/dweller | 56 |
| Chiroptera | Molossidae | *Mops leucostigma* | dweller | 63 |
| Chiroptera | Molossidae | *Mormopterus jugularis* | dweller | 63 |
| Chiroptera | Molossidae | *Mormopterus planiceps* | dweller | 64 |
| Chiroptera | Molossidae | *Nyctinomops aurispinosus* | visitor/dweller | 58 |
| Chiroptera | Molossidae | *Nyctinomops laticaudatus* | visitor/dweller | 59 |
| Chiroptera | Molossidae | *Nyctinomops macrotis* | visitor/dweller | 58 |
| Chiroptera | Molossidae | *Otomops martiensseni* | dweller | 65 |
| Chiroptera | Molossidae | *Promops centralis* | visitor/dweller | 59 |
| Chiroptera | Molossidae | *Promops nasutus* | visitor/dweller | 58 |
| Chiroptera | Molossidae | *Tadarida aegyptiaca* | dweller | 65 |
| Chiroptera | Molossidae | *Tadarida australis* | visitor/dweller | 66 |
| Chiroptera | Molossidae | *Tadarida brasiliensis* | dweller | 67 |
| Chiroptera | Molossidae | *Tadarida teniotis* | dweller | 68 |
| Chiroptera | Nycteridae | *Nycteris thebaica* | dweller | 69, 56 |
| Chiroptera | Phyllostomidae | *Artibeus cinereus* | visitor/dweller | 58 |
| Chiroptera | Phyllostomidae | *Artibeus lituratus* | dweller | 70, 163 |
| Chiroptera | Phyllostomidae | *Artibeus planirostris* | visitor/dweller | 58 |
| Chiroptera | Phyllostomidae | *Carollia perspicillata* | visitor/dweller | 58 |
| Chiroptera | Phyllostomidae | *Desmodus rotundus* | visitor/dweller | 71 |
| Chiroptera | Phyllostomidae | *Glossophaga soricina* | visitor/dweller | 58 |
| Chiroptera | Phyllostomidae | *Platyrrhinus lineatus* | visitor/dweller | 58 |
| Chiroptera | Phyllostomidae | *Sturnira lilium* | dweller | 72 |
| Chiroptera | Pteropodidae | *Cynopterus sphinx* | dweller | 73 |
| Chiroptera | Pteropodidae | *Eidolon helvum* | visitor | 74 |
| Chiroptera | Pteropodidae | *Epomophorus wahlbergi* | visitor/dweller | 75 |
| Chiroptera | Pteropodidae | *Pteropus alecto* | visitor | 76 |
| Chiroptera | Pteropodidae | *Pteropus giganteus* | visitor | 77 |
| Chiroptera | Pteropodidae | *Pteropus poliocephalus* | visitor | 78 |
| Chiroptera | Pteropodidae | *Pteropus scapulatus* | visitor | 78 |
| Chiroptera | Pteropodidae | *Rousettus aegyptiacus* | dweller | 79 |
| Chiroptera | Pteropodidae | *Rousettus leschenaultii* | dweller | 80 |
| Chiroptera | Rhinolophidae | *Rhinolophus ferrumequinum* | visitor | 81 |
| Chiroptera | Rhinolophidae | *Rhinolophus hipposideros* | visitor | 82 |
| Chiroptera | Rhinopomatidae | *Rhinopoma hardwickii* | visitor/dweller | 80 |
| Chiroptera | Vespertilionidae | *Chalinolobus gouldii* | dweller | 66 |
| Chiroptera | Vespertilionidae | *Eptesicus fuscus* | dweller | 83 |
| Chiroptera | Vespertilionidae | *Eptesicus nilssonii* | visitor/dweller | 84 |
| Chiroptera | Vespertilionidae | *Eptesicus serotinus* | dweller | 85 |
| Chiroptera | Vespertilionidae | *Hypsugo savii* | dweller | 68 |
| Chiroptera | Vespertilionidae | *Kerivoula picta* | dweller | 86 |
| Chiroptera | Vespertilionidae | *Lasiurus borealis* | visitor | 87 |
| Chiroptera | Vespertilionidae | *Myotis bocagii* | dweller | 69 |
| Chiroptera | Vespertilionidae | *Neoromicia nanus* | dweller | 88 |
| Chiroptera | Vespertilionidae | *Nyctalus lasiopterus* | visitor | 89 |
| Chiroptera | Vespertilionidae | *Nyctalus noctula* | dweller | 90, 164 |
| Chiroptera | Vespertilionidae | *Nyctalus plancyi* | dweller | 91 |
| Chiroptera | Vespertilionidae | *Pipistrellus abramus* | dweller | 91 |
| Chiroptera | Vespertilionidae | *Pipistrellus ceylonicus* | visitor | 80 |
| Chiroptera | Vespertilionidae | *Pipistrellus coromandra* | visitor/dweller | 80 |
| Chiroptera | Vespertilionidae | *Pipistrellus javanicus* | dweller | 92 |
| Chiroptera | Vespertilionidae | *Pipistrellus kuhlii* | dweller | 93 |
| Chiroptera | Vespertilionidae | *Pipistrellus pipistrellus* | dweller | 94 |
| Chiroptera | Vespertilionidae | *Pipistrellus pygmaeus* | dweller | 95 |
| Chiroptera | Vespertilionidae | *Pipistrellus tenuis* | dweller | 92 |
| Chiroptera | Vespertilionidae | *Plecotus auritus* | visitor/dweller | 96 |
| Chiroptera | Vespertilionidae | *Plecotus austriacus* | visitor/dweller | 97 |
| Chiroptera | Vespertilionidae | *Scotophilus dinganii* | visitor | 56 |
| Chiroptera | Vespertilionidae | *Scotophilus heathii* | visitor | 80 |
| Chiroptera | Vespertilionidae | *Scotophilus kuhlii* | visitor/dweller | 54 |
| Chiroptera | Vespertilionidae | *Vespertilio murinus* | visitor | 98 |
| Didelphimorphia | Didelphidae | *Didelphis virginiana* | dweller | 99 |
| Didelphimorphia | Didelphidae | *Monodelphis domestica* | visitor/dweller | 100 |
| Diprotodontia | Macropodidae | *Macropus giganteus* | visitor | 101, 165 |
| Diprotodontia | Petauridae | *Petaurus breviceps* | visitor | 102 |
| Diprotodontia | Phalangeridae | *Trichosurus vulpecula* | dweller | 103 |
| Diprotodontia | Pseudocheiridae | *Pseudocheirus peregrinus* | dweller | 104 |
| Eulipotyphla | Erinaceidae | *Atelerix frontalis* | dweller | 105 |
| Eulipotyphla | Erinaceidae | *Erinaceus europaeus* | dweller | 106 |
| Eulipotyphla | Erinaceidae | *Erinaceus roumanicus* | dweller | 107 |
| Eulipotyphla | Soricidae | *Blarina brevicauda* | dweller | 108 |
| Eulipotyphla | Soricidae | *Crocidura hirta* | dweller | 109 |
| Eulipotyphla | Soricidae | *Crocidura leucodon* | dweller | 110 |
| Eulipotyphla | Soricidae | *Crocidura russula* | dweller | 111 |
| Eulipotyphla | Soricidae | *Crocidura suaveolens* | dweller | 112 |
| Eulipotyphla | Soricidae | *Sorex araneus* | dweller | 111 |
| Eulipotyphla | Soricidae | *Sorex minutus* | dweller | 111 |
| Eulipotyphla | Soricidae | *Suncus etruscus* | dweller | 115 |
| Eulipotyphla | Soricidae | *Suncus murinus* | dweller | 113 |
| Hyracoidea | Procaviidae | *Procavia capensis* | dweller | 114 |
| Lagomorpha | Leporidae | *Oryctolagus cuniculus* | visitor/dweller | 115 |
| Lagomorpha | Leporidae | *Sylvilagus floridanus* | dweller | 116 |
| Peramelemorphia | Peramelidae | *Isoodon obesulus* | dweller | 116 |
| Peramelemorphia | Peramelidae | *Perameles nasuta* | dweller | 117 |
| Primates | Cebidae | *Callithrix jacchus* | dweller | 118 |
| Primates | Cebidae | *Callithrix kuhlii* | dweller | 119 |
| Primates | Cebidae | *Callithrix penicillata* | dweller | 120, 166 |
| Primates | Cebidae | *Saguinus bicolor* | dweller | 121 |
| Primates | Cebidae | *Saguinus geoffroyi* | dweller | 122 |
| Primates | Cebidae | *Saguinus leucopus* | dweller | 123 |
| Primates | Cercopithecidae | *Chlorocebus aethiops* | visitor | 124 |
| Primates | Cercopithecidae | *Chlorocebus pygerythrus* | dweller | 125 |
| Primates | Cercopithecidae | *Macaca fascicularis* | visitor/dweller | 126 |
| Primates | Cercopithecidae | *Macaca mulatta* | visitor | 127, 132, 23 |
| Primates | Cercopithecidae | *Macaca radiata* | visitor/dweller | 128 |
| Primates | Cercopithecidae | *Macaca sylvanus* | dweller | 129 |
| Primates | Cercopithecidae | *Papio anubis* | visitor | 130 |
| Primates | Cercopithecidae | *Papio ursinus* | visitor | 131 |
| Primates | Cercopithecidae | *Semnopithecus entellus* | dweller | 132, 167 |
| Rodentia | Caviidae | *Hydrochoerus hydrochaeris* | dweller | 133, 168 |
| Rodentia | Cricetidae | *Calomys musculinus* | dweller | 134 |
| Rodentia | Cricetidae | *Microtus arvalis* | dweller | 110 |
| Rodentia | Cricetidae | *Peromyscus leucopus* | dweller | 135 |
| Rodentia | Cricetidae | *Sigmodon fulviventer* | dweller | 136 |
| Rodentia | Cricetidae | *Sigmodon hispidus* | visitor/dweller | 137 |
| Rodentia | Hystricidae | *Hystrix cristata* | visitor/dweller | 138 |
| Rodentia | Muridae | *Apodemus agrarius* | dweller | 139, 110 |
| Rodentia | Muridae | *Apodemus flavicollis* | dweller | 110 |
| Rodentia | Muridae | *Apodemus sylvaticus* | dweller | 140 |
| Rodentia | Muridae | *Mastomys natalensis* | dweller | 141 |
| Rodentia | Muridae | *Mus musculus* | dweller | 142 |
| Rodentia | Muridae | *Rattus exulans* | dweller | 143 |
| Rodentia | Muridae | *Rattus flavipectus* | dweller | 45, 162 |
| Rodentia | Muridae | *Rattus norvegicus* | dweller | 144 |
| Rodentia | Muridae | *Rattus rattus* | dweller | 145 |
| Rodentia | Muridae | *Sundamys muelleri* | dweller | 146 |
| Rodentia | Myocastoridae | *Myocastor coypus* | dweller | 147 |
| Rodentia | Nesomyidae | *Cricetomys gambianus* | visitor/dweller | 109 |
| Rodentia | Sciuridae | *Callosciurus finlaysonii* | dweller | 148 |
| Rodentia | Sciuridae | *Funambulus pennantii* | dweller | 149 |
| Rodentia | Sciuridae | *Marmota monax* | dweller | 150 |
| Rodentia | Sciuridae | *Sciurus anomalus* | dweller | 151 |
| Rodentia | Sciuridae | *Sciurus carolinensis* | dweller | 152 |
| Rodentia | Sciuridae | *Sciurus granatensis* | dweller | 153 |
| Rodentia | Sciuridae | *Sciurus niger* | dweller | 149 |
| Rodentia | Sciuridae | *Sciurus vulgaris* | dweller | 154 |
| Rodentia | Sciuridae | *Tamias striatus* | dweller | 155 |

**Table S3.** Sample size for all datasets used, and respective number of urban species.

| **Urban** | **Taxon** | **N** | **N Urban** | **% Urban** |
| --- | --- | --- | --- | --- |
| Visitor | Carnivora | 73 | 8 | 11 |
|  | Cetartiodactyla | 68 | 8 | 11.8 |
|  | Primates | 130 | 4 | 3.1 |
|  | Chiroptera | 47 | 6 | 12.8 |
| Dweller | Carnivora | 92 | 15 | 16.3 |
|  | Rodentia | 202 | 19 | 9.4 |
|  | Primates | 132 | 6 | 4.5 |
|  | Eulipotyphla | 24 | 7 | 29.2 |
|  | Chiroptera | 52 | 11 | 21.1 |

**Fig. S1.** Relationship between the resolution of urban density and urban mammal richness raster maps and their corresponding Spearman’s correlation coefficient.

**References**

1.     Norberg, U. M., & Rayner, J. M. (1987). Ecological morphology and flight in bats (Mammalia; Chiroptera): wing adaptations, flight performance, foraging strategy and echolocation. Phil. Trans. R. Soc. Lond. B, 316(1179), 335-427.

2.     Jones, G., Morton, M., Hughes, P. M., & Budden, R. M. (1993). Echolocation, flight morphology and foraging strategies of some West African hipposiderid bats. Journal of Zoology, 230(3), 385-400.

3.     Norberg U. M., & Fenton M. B. (1988). Carnivorous bats?. Biological Journal of the Linnean Society, 33(4), 383-394.

4.     Roslan, A. (2014). Wing loading and aspect ratio of Roundleaf bats (Family: Hipposideridae). Bachelor Science Thesis. University Malaysia, Sarawak.

5.     Sahley, C. T., Horner, M. A., & Fleming, T. H. (1993). Flight Speeds and Mechanical Power Outputs of the Nectar-Feedint Bat, Leptonycteris curasoae (Phyllostomidae: Glossophaginae). Journal of Mammalogy, 74(3), 594-600.

6.     McKenzie, N. L., & Rolfe, J. K. (1986). Structure of bat guilds in the Kimberley mangroves, Australia. The Journal of Animal Ecology, 401-420.

7.     Jennings, N. V., Parsons, S., Barlow, K. E., & Gannon, M. R. (2004). Echolocation calls and wing morphology of bats from the West Indies. Acta Chiropterologica, 6(1), 75-90.

8.     Bullen, R. D., & McKenzie, N. L. (2007). Bat wing airfoil and planform structures relating to aerodynamic cleanliness. Australian Journal of Zoology, 55(4), 237-247.

9.     Plumpton, D. L., & Jones, J. K. (1992). Rhynchonycteris naso. Mammalian Species, (413), 1-5.

10.  McKenzie, N. L., Gunnell, A. C., Yani, M., & Williams, M. R. (1995). Correspondence between plight morphology and foraging ecology in some palaeotropical bats. Australian Journal of Zoology, 43(3), 241-257.

11.  Bullen, R. D., & McKenzie, N. L. (2004). Bat flight-muscle mass: implications for foraging strategy. Australian Journal of Zoology, 52(6), 605-622.

12.  Zhang, L., Liang, B., Parsons, S., Wei, L., & Zhang, S. (2007). Morphology, echolocation and foraging behaviour in two sympatric sibling species of bat (Tylonycteris pachypus and Tylonycteris robustula)(Chiroptera: Vespertilionidae). Journal of Zoology, 271(3), 344-351.

13.  Raichev, E. G., Tsunoda, H., Newman, C., Masuda, R., Georgiev, D. M., & Kaneko, Y. (2013). The Reliance of the golden jackal (*Canis* *aureus*) on anthropogenic foods in winter in Central Bulgaria. Mammal Study, 38(1), 19-27.

14.  Gese, E.M. & Bekoff, M. (2004). Coyote Canis latrans say, 1823. In Canids: foxes, wolves, jackals and dogs. Status survey and conservation action plan: 8187. Sillero-Zubiri, C., Hoffmann, M., Macdonald, D.W. & IUCN/SSC Canid Specialist Group (Eds). Cambridge: IUCN Publications Services

15.  Bateman, P. W., & Fleming, P. A. (2012). Big city life: carnivores in urban environments. Journal of Zoology, 287(1), 1-23.

16.  Drygala, F., Stier, N., Zoller, H., Boegelsack, K., Mix, H. M., & Roth, M. (2008). Habitat use of the raccon dog (*Nyctereutes procyonoides*) in north-eastern Germany. Mammalian Biology-Zeitschrift fur Saugetierkunde, 73(5), 371-378.

17.  Harrison, R.L. (1997). A comparison of gray fox ecology between residential and undeveloped rural landscapes. J. Wildl. Mgmt. 61, 112-122.

18.  Wilkinson, D. & Smith, G.C. (2001). A preliminary survey for changes in urban Fox (*Vulpes vulpes*) densities in England and Wales, and implication for rabies control. Mammal Rev. 31, 107-110.

19.  Schmidt, P.M., Lopez, R.R. & Pierce, B.L. (2007). Estimating free-roaming cat densities in urban areas: comparison of mark-resight and distance sampling. Wildl. Biol. Pract. 3, 18-27.

20.  Athreya, V., Odden, M., Linnell, J. D., Krishnaswamy, J., & Karanth, K. U. (2016). A cat among the dogs: leopard *Panthera pardus* diet in a human-dominated landscape in western Maharashtra, India.Oryx, 50(01), 156-162.

21.  Benson, J. F., Sikich, J. A., & Riley, S. P. (2016). Individual and population level resource selection patterns of mountain lions preying on mule deer along an urban-wildland gradient. PloS one, 11(7), e0158006.

22.  Freitag, S., Hobson, C., Biggs, H. C., & Van Jaarsveld, A. S. (1998, May). Testing for potential survey bias: the effect of roads, urban areas and nature reserves on a southern African mammal data set. In Animal Conservation forum (Vol. 1, No. 2, pp. 119-127). Cambridge University Press.

23.  Gosai, K. R., Koju, N. P., Karmacharya, D. K., & Basukala, S. Conflict between Humans and Urban Wild-life in Bhaktapur.

24.  Gorman, M. L. (1975). The diet of feral *Herpestes auropunctatus* (Carnivora: Viverridae) in the Fijian Islands. Journal of Zoology, 175(2), 273-278.

25.  Hays, W. S., & Conant, S. (2007). Biology and impacts of Pacific Island invasive species. 1. A worldwide review of effects of the small Indian mongoose, *Herpestes javanicus* (Carnivora: Herpestidae). Pacific Science, 61(1), 3-16.

26.  Yirga, G., Ersino, W., De Iongh, H. H., Leirs, H., Gebrehiwot, K., Deckers, J., & Bauer, H. (2013). Spotted hyena (*Crocuta crocuta*) coexisting at high density with people in Wukro district, northern Ethiopia. Mammalian Biology- *Zeitschrift für Säugetierkunde*, 78(3), 193-197

27.  Abi-Said, M. R., & Abi-Said, D. M. (2007). Distribution of the Striped Hyaena (*Hyaena hyaena syriaca* Matius, 1882)(Carnivora: Hyaenidae) in urban and rural areas of Lebanon. Zoology in the Middle East, 42(1), 3-14.

28.  Rosatte, R.C., Power, M.J. & Macinnes, C.D. (1991). Ecology of urban skunks, raccoons, and foxes in metropolitan Toronto. In Wildlife conservation in metropolitan environ- ments. National institute for urban wildlife symposium series 2: 31-38. Adams, L.W. & Leedy, D.L. (Eds). Columbia

29.  Clark, K. D. (1994, February). Managing raccoons, skunks, and opossums in urban settings. In Proceedings of the Sixteenth Vertebrate Pest Conference (1994) (p. 10)

30.  Gallant, D, L Vasseur, M Dumond, R Trambley, and CH Berube. 2009. Habitat selection by river otters (*Lontra canadensis*) under contrasting land-use regimes. Canadian Journal of Zoology 87:422-432

31.  White, S., O’Neill, D., O’Meara, D. B., Shores, C., O’Reilly, C., Harrington, A. P., ... & Sleeman, D. P. (2013). A Non-Invasive Genetic Survey Of Otters (*Lutra lutra*). An Urban Environment: A Pilot Study With Citizen Scientists. IUCN Otter Spec. Group Bull, 30(2), 103-111.

32.  Theng, M., & Sivasothi, N. (2016). The Smooth-Coated Otter *Lutrogale perspicillata* (Mammalia: Mustelidae) in Singapore: Establishment and Expansion in Natural and Semi-Urban Environments. IUCN Otter Spec. Group Bull, 33(1), 37-49

33.  Delibes, M. (1983). Interspecific competition and the habitat of the stone marten *Martes foina* (Erxleben 1777) in Europe. Acta Zool. Fenn. 174, 229-231.

34.  Davison, J., Huck, M., Delahay, R.J. & Roper, T.J. (2009). Restricted ranging behaviour in a high-density population of urban badgers. J. Zool. (Lond.) 277, 45-53.

35.  Alcock, I., & Warsop, P. (1982). Diet, distribution and habitat preferences of stoats and weasels in Sheffield. Sorby Record, 20, 5-10.

36.  Stapleton, H. M., Dodder, N. G., Kucklick, J. R., Reddy, C. M., Schantz, M. M., Becker, P. R., Gulland, F., Porter, B. J., Wise, S. A. (2006). Determination of HBCD, PBDEs and MeO-BDEs in California sea lions (*Zalophus californianus*) stranded between 1993 and 2003. Marine Pollution Bulletin, 52(5), 522-531.

37.  Nickel, B. A., Grigg, E. K., Green, D. E., Allen, S., & Markowitz, H. (2001). Pacific harbor seal (*Phoca vitulina richardsi*) distribution, movement, and foraging activities within an urban estuary: implications for the effects of seismic retroffiting in San Franciso Bay, California. In Abstract 14th Biennial Conference on the Biology of Marine Mammals, Vancouver, Canada Nov.

38.  Alves-Costa, C. P., Da Fonseca, G. A., & Christofaro, C. (2004). Variation in the diet of the brown-nosed coati (*Nasua nasua*) in southeastern Brazil. Journal of Mammalogy, 85(3), 478-482.

39.  Hadidian, J., Prange, S., Rosatte, R., Riley, S. P. D., & Gehrt, S. D. (2010). Raccoons (*Procyon lotor*). Urban carnivores: ecology, conflict, and conservation (SD Gehrt, SPD Riley, and BL Cypher, eds.). Johns Hopkins University Press, Baltimore, Maryland, 35-47.

40.  Karanth, K. U., & Chellam, R. (2009). Carnivore conservation at the crossroads. Oryx, 43(1), 1.

41.  Beckmann, J. P., & Lackey, C. W. (2008). Carnivores, urban landscapes, and longitudinal studies: a case history of black bears.

42.  Widdows, C. D., Ramesh, T., & Downs, C. T. (2015). Factors affecting the distribution of large spotted genets (*Genetta tigrina*) in an urban environment in South Africa. Urban ecosystems, 18(4), 1401-1413.

43.  Saito, M., & Koike, F. (2013). Distribution of wild mammal assemblages along an urban-rural-forest landscape gradient in warm-temperate East Asia. PloS one, 8(5), e65464.

44.  Yasuma, S., & Andau, M. (2000). Mammals of Sabah, Part 2, habitat and ecology. Japan International Cooperation Agency and Sabah Wildlife Department, Kota Kinabalu, Sabah, Malaysia.

45.  Kait, R., Sahi, D.N.: Determination of the local, national/global status and effect of urbanization on Carnivora mammals in Jammu District and Trikuta Hills of Jandk, India. International Journal of Biodiversity and Conservation, 4(14), 530-534 (2012)

46.  Moriarty, A. (2004). The liberation, distribution, abundance and management of wild deer in Australia. Wildlife Research, 31(3), 291-299.

47.  Dickman, C. R. (1987). Habitat fragmentation and vertebrate species richness in an urban environment. Journal of Applied Ecology, 337-351.

48.  Frair, J. L., Merrill, E. H., Visscher, D. R., Fortin, D., Beyer, H. L., & Morales, J. M. (2005). Scales of movement by elk (*Cervus elaphus*) in response to heterogeneity in forage resources and predation risk. Landscape Ecology, 20(3), 273-287.

49.  Duarte, J., Farfán, M. A., Fa, J. E., & Vargas, J. M. (2015). Deer populations inhabiting urban areas in the south of Spain: habitat and conflicts. *European Journal of Wildlife Research*, *61*(3), 365-377.

50.  McCleery, R. (2010). Urban mammals. Urban ecosystem ecology, (urban ecosystem), 87-102.

51.  Massei, G., Kindberg, J., Licoppe, A., Gacic, D., Šprem, N., Kamler, J., Baubet, E., Hohmann, U., Monaco, A., Ozoli, J., Cellina, S., Podgórski, T., Fonseca, C., Markov, N., Pokorny, B., Rosell, C., Náhlik A. (2015). Wild boar populations up, numbers of hunters down? A review of trends and implications for Europe. Pest management science, 71(4), 492-500.

52.  Ticer, C. L., Morrell, T. E., & Devos Jr, J. C. (2001). Diurnal bed-site selection of urban-dwelling javelina in Prescott, Arizona. The Journal of wildlife management, 136-140.

53.  Keuroghlian, A., Eaton, D. P., & Longland, W. S. (2004). Area use by white-lipped and collared peccaries (*Tayassu pecari* and *Tayassu* *tajacu*) in a tropical forest fragment. Biological Conservation, 120(3), 411-425.

54.  Pottie, S. A., Lane, D. J., Kingston, T., & Y.-H. Lee, B. P. (2005). The microchiropteran bat fauna of Singapore. Acta Chiropterologica, 7(2), 237-247.

55.  Fenton, M. B., & Eger, J. L. (2002). *Chaerephon chapini*. Mammalian Species, 1-2.

56.  Schoeman, M. C. (2016). Light pollution at stadiums favors urban exploiter bats. Animal Conservation, 19(2), 120-130.

57.  Thong, V. D. (2015). Taxonomic and distributional assessments of *Chaerephon plicatus* (Chiroptera: Molossidae) from Vietnam. Tap Chi Sinh Hoc, 36(4), 479-486.

58.  Nunes, H., Rocha, F. L., & Cordeiro-Estrela, P. (2017). Bats in urban areas of Brazil: roosts, food resources and parasites in disturbed environments. Urban Ecosystems, 20(4), 953-969.

59.  Jung, K., & Kalko, E. K. (2011). Adaptability and vulnerability of high flying Neotropical aerial insectivorous bats to urbanization. Diversity and Distributions, 17(2), 262-274.

60.  Sodrè, M. M., da Rosa, A. R., Gregorin, R., & Guimaràes, M. M. (2008). Range extension for Thomas’ Mastiff bat *Eumops maurus* (Chiroptera: Molossidae) in northern, central and southeastern Brazil. Revista Brasileira de Zoologia, 25, 379-382.

61.  Oprea, M., Mendes, P., Vieira, T. B., & Ditchfield, A. D. (2009). Do wooded streets provide connectivity for bats in an urban landscape?. Biodiversity and Conservation, 18(9), 2361-2371.

62.  Esbrard, C. E., Jesus, A. C., Motta, A. G., Bergallo, H. G., & Gettinger, D. (2005). *Hesperoctenes fumarius* (Hemiptera: Polyctenidae) infesting *Molossus rufus* (Chiroptera: Molossidae) in southeastern Brazil. Journal of Parasitology, 91(2), 465-467.

63.  Lopez-Baucells, A., Rocha, R., Forbes, K. M., & Cabeza, M. (2017). Roost selection by synanthropic bats in rural Madagascar: what makes non-traditional structures so tempting?. Hystrix, the Italian Journal of Mammalogy, 28, 28-35.

64.  Scanlon, A., & Petit, S. T. (2007). The diet and activity of urban insectivorous bats and recommendations for habitat management in the city of Adelaide. South Australian Naturalist, 81(1).

65.  Schoeman, M. C. (2016). Light pollution at stadiums favors urban exploiter bats. Animal Conservation, 19(2), 120-130.

66.  Luck, G. W., Smallbone, L., Threlfall, C., & Law, B. (2013). Patterns in bat functional guilds across multiple urban centres in south-eastern Australia. Landscape ecology, 28(3), 455-469.

67.  Avila-Flores, R., & Fenton, M. B. (2005). Use of spatial features by foraging insectivorous bats in a large urban landscape. Journal of Mammalogy, 86(6), 1193-1204.

68.  Russo, D., & Ancillotto, L. (2015). Sensitivity of bats to urbanization: a review. Mammalian Biology-Zeitschrift fur Saugetierkunde, 80(3), 205-212.

69.  Naidoo, S., Mackey, R. L., & Schoeman, M. C. (2011). Foraging ecology of insectivorous bats (Chiroptera) at a polluted and an unpolluted river in an urban landscape. Durban Museum Novitates, 34, 21-28.

70.  Sazima, I., Fischer, W. A., Sazima, M., & Fischer, E. A. (1994). The fruit bat *Artibeus lituratus* as a forest and city dweller. Ciencia e cultura(Sao Paulo), 46(3), 164-168.

71.  Uieda, W. (1995). The common vampire bat in urban environments from Southeastern Brazil. Chiroptera Neotropical, 1(2), 22-24.

72.  Montes, M. A., de Figueiredo, D., Miller, B. G., & Leal, E. S. B. (2015). Fluctuating asymmetry in populations of bats: species adapted to urban environments are not hampered by habitat degradation. Chiroptera Neotropical, 21(1), 1305-1311.

73.  Corlett, R. T. (2005). Interactions between birds, fruit bats and exotic plants in urban Hong Kong, South China. Urban Ecosystems, 8(3), 275-283.Corlett, R. T. (2005). Interactions between birds, fruit bats and exotic plants in urban Hong Kong, South China. Urban Ecosystems, 8(3), 275-28

74.  Baker, K. S., Todd, S., Marsh, G. A., Crameri, G., Barr, J., Kamins, A. O., Peel, A. J., Yu, M., Hayman, D. T. S., Nadjm, B., Mtove, G., Amos, B., Reyburn, H., Nyarko, E., Suu-Ire, R., Murcia, P. R., Cunningham, A. A., Wood, J. L. N., Wang, L. F. (2013). Novel, potentially zoonotic paramyxoviruses from the African straw-colored fruit bat *Eidolon helvum*. Journal of virology, 87(3), 1348-1358.

75.  Rollinson, D. P., Coleman, J. C., & Downs, C. T. (2014). Roost temperature and fidelity of Wahlberg's epauletted fruit bat, *Epomophorus* *wahlbergi*, in an urban environment. African Zoology, 49(2), 173-180.

76.  Markus, N., & Hall, L. (2004). Foraging behaviour of the black flying-fox (*Pteropus alecto*) in the urban landscape of Brisbane, Queensland. Wildlife Research, 31(3), 345-355.

77.  Mahmood-ul-Hassan, M., Gulraiz, T. L., Rana, S. A., & Javid, A. (2010). The diet of Indian flying-foxes (*Pteropus giganteus*) in urban habitats of Pakistan. Acta Chiropterologica, 12(2), 341-347.

78.  Parris, K. M., & Hazell, D. L. (2005). Biotic effects of climate change in urban environments: The case of the grey-headed flying-fox (*Pteropus poliocephalus*) in Melbourne, Australia. Biological Conservation, 124(2), 267-276.

79.  Barclay, R. M. R., & Jacobs, D. S. (2011). Differences in the foraging behaviour of male and female Egyptian fruit bats (*Rousettus* *aegyptiacus*). Canadian Journal of Zoology, 89(6), 466-473.

80.  Elangovan, V., & Kumar, M. (2015). Diversity, Roost Selection and Ecological Importance of the Bats of Uttar Pradesh.Uttar Pradesh State Biodiversity Board.

81.  Ransome, R., & Hutson, A. M. (2000). Action plan for the conservation of the greater horseshoe bat in Europe (*Rhinolophus* *ferrumequinum*) (No. 18-104). Council of Europe.

82.  Boldogh, S., Dobrosi, D., & Samu, P. (2007). The effects of the illumination of buildings on house-dwelling bats and its conservation consequences. Acta Chiropterologica, 9(2), 527-534.

83.  Agosta, S. J. (2002). Habitat use, diet and roost selection by the big brown bat (*Eptesicus fuscus*) in North America: a case for conserving an abundant species. Mammal Review, 32(3), 179-198.

84.  Haupt M, Menzler S, Schmidt S (2006) Flexibility of habitat use in Eptesicus nilssonii: does the species pro t from anthropogenically altered habitats? J Mammal 87(2):351-361

85.  Catto, C. M. C., Hutson, A. M., Raccey, P. A., & Stephenson, P. J. (1996). Foraging behaviour and habitat use of the serotine bat (*Eptesicus serotinus*) in southern England. Journal of Zoology, 238(4), 623-633.

86.  Madhavan, A., Rajasekar, S., & Isaac, S. S. (2014). Foliage roosting by the painted bat, *Kerivoula picta* in Thrissur, Kerala. Small Mammal Mail, 5, 14.

87.  Walters, B. L., Ritzi, C. M., Sparks, D. W., & Whitaker Jr, J. O. (2007). Foraging behavior of eastern red bats (*Lasiurus borealis*) at an urban-rural interface. The American midland naturalist, 157(2), 365-373.

88.  Naidoo, S., Vosloo, D., & Schoeman, M. C. (2013). Foraging at wastewater treatment works increases the potential for metal accumulation in an urban adapter, the banana bat (*Neoromicia nana*). African Zoology, 48(1), 39-55.

89.  Popa_Lisseanu, A. G., Bontadina, F., & Ibáñez, C. (2009). Giant noctule bats face conflicting constraints between roosting and foraging in a fragmented and heterogeneous landscape. Journal of zoology, 278(2), 126-133.

90.  Bihari, Z., & Bakos, J. (2001). Roost selection of *Nyctalus noctula* (Chiroptera, Vespertilionidae) in urban habitat. Proceedings of the Vlllth EBRS, 2.

91.  Heiker et al. 2018. Mercury Bioaccumulation in Two Species of Insectivorous Bats from Urban China: Influence of Species, Age, and Land Use Type. Archives of Environmental Contamination and Toxicology *75*(4), 585-593

92.  Sajid Nadeem, M., Zafar, S., Rashid Kayani, A., Mushtaq, M., Azhar Beg, M., & Farooq Nasir, M. (2013). Distribution and Roosting Habitats of Some Microchiropteran Bats in Rawalpindi District, Pakistan. Pakistan Journal of Zoology, 45(2).

93.  Ancillotto, L., Tomassini, A., & Russo, D. (2016). The fancy city life: Kuhl’s pipistrelle, *Pipistrellus kuhlii*, benefits from urbanisation. Wildlife Research, 42(7), 598-606.

94.  Duchamp, J. E., Sparks, D. W., & Whitaker, Jr, J. O. (2004). Foraging-habitat selection by bats at an urban-rural interface: comparison between a successful and a less successful species. Canadian Journal of Zoology, 82(7), 1157-1164.

95.  Davidson-Watts, I., Walls, S., & Jones, G. (2006). Differential habitat selection by *Pipistrellus pipistrellus* and *Pipistrellus pygmaeus* identifies distinct conservation needs for cryptic species of echolocating bats. Biological conservation, 133(1), 118-127.

96.  Entwistle, A. C., Racey, P. A., & Speakman, J. R. (1997). Roost selection by the brown long-eared bat *Plecotus auritus*. Journal of Applied Ecology, 399-408.

97.  Stoycheva, S., Georgiev, D., Pandourski, I., & Tilova, E. (2009). Bat diversity in two large towns of the Upper Thrace, Bulgaria (Chiroptera). Lynx, ns (Praha), 40, 83-93.

98.  Rydell, J., & Baag¿e, H. J. (1994). *Vespertilio murinus*. Mammalian species, (467), 1-6.

99.  Wright, J. D., Burt, M. S., & Jackson, V. L. (2012). Influences of an urban environment on home range and body mass of Virginia opossums (*Didelphis virginiana*). Northeastern Naturalist, 19(1), 77-86.

100.  Eisenberg, J., K. Redford. 1999. Mammals of the Neotropics: The Central Neotropics, Vol. 3. Chicago, IL: The University of Chicago Press.

101.  Tribe, A., Hanger, J., McDonald, I. J., Loader, J., Nottidge, B. J., McKee, J. J., & Phillips, C. J. (2014). A reproductive management program for an urban population of eastern grey kangaroos (*Macropus giganteus*). Animals, 4(3), 562-582.

102.  Caryl, F. M., Thomson, K., & Ree, R. (2013). Permeability of the urban matrix to arboreal gliding mammals: Sugar gliders in Melbourne, Australia. Austral Ecology, 38(6), 609-616.

103.  Eymann, J. U. T. T. A., Herbert, C. A., & Cooper, D. W. (2006). . Management issues of urban common brushtail possums *Trichosurus* *vulpecula*: a loved or hated neighbour. Australian Mammalogy, 28(2), 153-171.

104.  Temby, I. D. (2004, May). Urban wildlife issues in Australia. In Proceedings of the 4th International Symposium on Urban Wildlife Conservation.(Eds WW Shaw, LK Harris and L. Vandruff.) pp (pp. 26-34).

105.  Light, J. L. (2016). The distribution and urban occurrence of the elusive Southern African hedgehog (*Atelerix frontalis*) (Doctoral dissertation).

106. Hubert, P., Julliard, R., Biagianti, S., & Poulle, M. L. (2011). Ecological factors driving the higher hedgehog (*Erinaceus europeaus*) density in an urban area compared to the adjacent rural area. Landscape and Urban Planning, 103(1), 34-43.

107. Földvári, G., Rigó, K., Jablonszky, M., Biró, N., Majoros, G., Molnár, V., & Tóth, M. (2011). Ticks and the city: Ectoparasites of the Northern white-breasted hedgehog (*Erinaceus roumanicus*) in an urban park. Ticks Tick Borne Dis 2011; 2(4): 231-234.

108. Brack Jr, V. (2006). Short-tailed Shrews (*Blarina brevicauda*) exhibit unusual behavior in an urban environment. Urban Hab, 4, 127-132.

109. Durnez, L., Eddyani, M., Mgode, G. F., Katakweba, A., Katholi, C. R., Machang'u, R. R., ... & Leirs, H. (2008). First detection of mycobacteria in African rodents and insectivores, using stratified pool screening. Applied and Environmental Microbiology, 74(3), 768-773.

110. Lopucki, R., & Kitowski, I. (2017). How small cities affect the biodiversity of ground-dwelling mammals and the relevance of this knowledge in planning urban land expansion in terms of urban wildlife. Urban Ecosystems, 20(4), 933-943.

111. Vergnes, A., Kerbiriou, C., & Clergeau, P. (2013). Ecological corridors also operate in an urban matrix: a test case with garden shrews. Urban ecosystems, 16(3), 511-525.

112. Gippoliti, S., & Amori, G. (2006). Historical data on non-volant mammals in Rome: What do they say about urban environment. Aldrovandia, 2, 69-72.

113. Sorace, A. (2001). Value to wildlife of urban-agricultural parks: a case study from Rome urban area. Environmental Management, 28(4), 547-560.

114. Wiid, R. E., & Butler, H. J. (2015). Population management of rock hyraxes (*Procavia capensis*) in residential areas. Pest management science, 71(2), 180-188.

115. Baker, P. J., & Harris, S. (2007). Urban mammals: what does the future hold? An analysis of the factors affecting patterns of use of residential gardens in Great Britain. Mammal Review, 37(4), 297-315.

116. Garden, J. G., Mcalpine, C. A., Possingham, H. P., & Jones, D. N. (2007). Habitat structure is more important than vegetation composition for local-level management of native terrestrial reptile and small mammal species living in urban remnants: A case study from Brisbane, Australia. *Austral Ecology*, *32*(6), 669-685.

117. Scott, L. K., Hume, I. D., & Dickman, C. R. (1999). Ecology and population biology of long-nosed bandicoots (*Perameles nasuta*) at North Head, Sydney Harbour National Park. Wildlife Research, 26(6), 805-821.

118.  Cunha, A. A., Vieira, M. V., & Grelle, C. E. (2006). Preliminary observations on habitat, support use and diet in two non-native primates in an urban Atlantic forest fragment: The capuchin monkey (*Cebus* sp.) and the common marmoset (*Callithrix jacchus*) in the Tijuca forest, Rio de Janeir

119. Rodrigues, N. N., & Martinez, R. A. (2014). Wildlife in our backyard: interactions between Wied's marmoset *Callithrix kuhlii* (Primates: Callithrichidae) and residents of Ilhus, Bahia, Brazil. Wildlife Biology, 20(2), 91-96.

120. Teixeira, B., Hirsch, A., Goulart, V. D., Passos, L., Teixeira, C. P., James, P., & Young, R. (2016). Good neighbours: distribution of black-tufted marmoset (*Callithrix penicillata*) in an urban environment. Wildlife Research, 42(7), 579-589.

121.  Gordo, M., Calleia, F. O., Vasconcelos, S. A., Leite, J. J., & Ferrari, S. F. (2013). The challenges of survival in a concrete jungle: conservation of the pied tamarin (*Saguinus bicolor*) in the urban landscape of Manaus, Brazil. In Primates in fragments (pp. 357-370). Springer New York.

122.  McNaughton, C. (2015). Characteristics of Geoffroy’s tamarin (*Saguinus geoffroyi*) population, demographics, and territory sizes in urban park habitat (Parque Natural Metropolitano, Panama City, Panama). Independent Study Project 2276, Ohio Wesleyan University

123. Poveda, K., & S‡nchez-Palomino, P. (2004). Habitat use by the white-footed tamarin, Saguinus leucopus: a comparison between a forest-dwelling group and an urban group in Mariquita, Colombia. Neotropical Primates, 12(1), 6-9.

124. Saj, T., Sicotte, P., & Paterson, J. D. (1999). Influence of human food consumption on the time budget of vervets. International Journal of Primatology, 20, 977-994

125. Mikula, P., Šaffa, G., Nelson, E., & Tryjanowski, P. (2017). Risk perception of vervet monkeys *Chlorocebus pygerythrus* to humans in urban and rural environments. Behavioural processes 147: 21-27.

126. Sussman, R. W., Shaffer, C. A., & Guidi, L. (2011). *Macaca fascicularis* in Mauritius: implications for macaque-human interactions and for future research on long-tailed macaques. Monkeys on the edge: ecology and management of long-tailed macaques and their interface with humans. Cambridge University Press.

127. Jaman, M. F., & Huffman, M. A. (2013). The effect of urban and rural habitats and resource type on activity budgets of commensal rhesus macaques (*Macaca mulatta*) in Bangladesh. Primates, 54(1), 49-59.

128. Singh, M., & Vinathe, S. (1990). Inter-population differences in the time budgets of bonnet monkeys (*Macaca radiata*). Primates, 31(4), 589-596.

129. Schurr, M. R., Fuentes, A., Luecke, E., Cortes, J., & Shaw, E. (2012). Intergroup variation in stable isotope ratios reflects anthropogenic impact on the Barbary macaques (*Macaca sylvanus*) of Gibraltar. Primates, 53(1), 31-40.

130. Quick, D. L. (1986). Activity budgets and the consumption of human food in two troops of baboons, *Papio anubis* at Gilgil, Kenya. Primate Ecology and Conservation. Cambridge University Press, Cambridge, 221-228.

131. Kaplan, B. S., O’Riain, M. J., van Eeden, R., & King, A. J. (2011). A low-cost manipulation of food resources reduces spatial overlap between baboons (*Papio ursinus*) and humans in conflict. International Journal of Primatology, 32(6), 1397-1412.

132.  Pirta, R. S. (1982). Conservation note: socioecology and conservation of macaques and langurs in Varanasi, India. American Journal of Primatology, 2(4), 401-403.

133. Queirogas, V. L., Del Claro, K., Nascimento, A. R. T., & Szabó, M. P. J. (2012). Capybaras and ticks in the urban areas of Uberlandia, Minas Gerais, Brazil: ecological aspects for the epidemiology of tick-borne diseases. Experimental and applied acarology, 57(1), 75-82.

134. Chiappero, M. B., Panzetta-Dutari, G. M., G—mez, D., Castillo, E., Polop, J. J., & Gardenal, C. N. (2011). Contrasting genetic structure of urban and rural populations of the wild rodent *Calomys musculinus* (Cricetidae, Sigmodontinae). Mammalian Biology-*für Säugetierkunde*, 76(1), 41-50.

135. Barko, V. A., Feldhamer, G. A., Nicholson, M. C., & Davie, D. K. (2003). Urban habitat: a determinant of white-footed mouse (*Peromyscus* *leucopus*) abundance in southern Illinois. Southeastern Naturalist, 2(3), 369-376.

136. Bock, C. E., Jones, Z. F., & Bock, J. H. (2006). Rodent communities in an exurbanizing southwestern landscape (USA). Conservation Biology, 20(4), 1242-1250.

137. Schroder, G. D., & Hulse, M. I. C. H. A. E. L. (1979). Survey of rodent populations associated with an urban landfill. American journal of public health, 69(7), 713-715.

138. Grano, M. (2016). An unusual urban refuge for the crested porcupine, *Hystrix cristata* (Linnaeus, 1758)(Mammalia Rodentia): the ancient Catacombs of Priscilla in Rome (Italy). Biodiversity Journal, 7, 345-346.

139. Luniak, M. (2004, May). Synurbization-adaptation of animal wildlife to urban development. In Proc. 4th Int. Symposium Urban Wildl. Conserv. Tucson (pp. 50-55).

140. Dickman, C. R., & Doncaster, C. P. (1989). The ecology of small mammals in urban habitats. II. Demography and dispersal. The Journal of Animal Ecology, 119-127.

141. Mercier, A., Garba, M., Bonnabau, H., Kane, M., Rossi, J. P., Dard, M. L., & Dobigny, G. (2013). Toxoplasmosis seroprevalence in urban rodents: a survey in Niamey, Niger. Memorias do Instituto Oswaldo Cruz, 108(4), 399-407.

142.  Pocock, M. J., Searle, J. B., & White, P. C. (2004). Adaptations of animals to commensal habitats: population dynamics of house mice *Mus* *musculus domesticus* on farms. Journal of Animal Ecology, 73(5), 878-888.

143. Bramley, G. N. (2014). Habitat use by kiore (*Rattus* *exulans*) and Norway rats (*R. norvegicus*) on Kapiti Island, New Zealand. New Zealand Journal of Ecology, 64-75.

144. Gardner-Santana, L. C., Norris, D. E., Fornadel, C. M., Hinson, E. R., Klein, S. L., & Glass, G. E. (2009). Commensal ecology, urban landscapes, and their influence on the genetic characteristics of city_dwelling Norway rats (*Rattus norvegicus*). Molecular ecology, 18(13), 2766-2778

145. Ervynck, A. (2002). Sedentism or urbanism? On the origin of the commensal black rat (*Rattus rattus*). Bones and the man. Studies in honour of Don Brothwell, Oxbow: Oxford, 95-109.

146. Wells, K., Lakim, M. B., & O’Hara, R. B. (2014). Shifts from native to invasive small mammals across gradients from tropical forest to urban habitat in Borneo. Biodiversity and conservation, 23(9), 2289-2303.

147.  Meyer, J., Klemann, N., & Halle, S. (2005). Diurnal activity patterns of coypu in an urban habitat. Acta theriologica, 50(2), 207-211.

148.  Bertolino, S., & Lurz, P. W. (2013). *Callosciurus* squirrels: worldwide introductions, ecological impacts and recommendations to prevent the establishment of new invasive populations. Mammal Review, 43(1), 22-33.

149. Palmer, G. H., Koprowski, J., & Pernas, T. (2007). Tree squirrels as invasive species: conservation and management implications. In: Witmer, G. W., W. C. Pitt, and K. A. Fagerstone (eds). Managing vertebrate invasive species: Proceedings of an international symposium. USDA/APHIS Wildlife Services, National Wildlife Research Center, Fort Collins, Colorado, USA

150. Lehrer, E. W., Fredebaugh, S. L., Schooley, R. L., & Mateus-Pinilla, N. E. (2010). Prevalence of antibodies to *Toxoplasma gondii* in woodchucks across an urban-rural gradient. Journal of Wildlife Diseases, 46(3), 977-980.

151. Osborn, D. J. (1964). The hare, porcupine, beaver, squirrels, jerboas, and dormice of Turkey. Mammalia, 28(4), 573-592.

152.  Shorten, M. (1946). A survey of the distribution of the American grey squirrel (*Sciurus carolinensis*) and the British red squirrel (S. vulgaris leucourus) in England and Wales in 1944-5. Journal of Animal Ecology, 15(1), 82-92.

153. Heaney, L., R. Thorington, Jr.. 1978. Ecology of Neotropical Red-Tailed Squirrels, *Sciurus granatensis*, in the Panama Canal Zone. Journal of Mammalogy, 59(4): 846-851.

154. Babińska-Werka, J., & Żółw, M. (2008, August). Urban populations of the red squirrel (*Sciurus vulgaris*) in Warsaw. In Annales Zoologici Fennici (Vol. 45, No. 4, pp. 270-276). Finnish Zoological and Botanical Publishing.

155. Weckel, M. A. R. K., & Giuliano, W. (2001). Forest structure in urban parks: effects on eastern chipmunk distribution. Northeast Wildlife, 56, 49-56.

156. Gehrt, S.D., Anchor, C. & White, L.A. (2009). Home range and landscape use of coyotes in a metropolitan landscape: conflict or coexistence? J. Mammal. 90, 1045-1057.

157. Harris, S. & Rayner, J.M.V. (1986b). Urban fox (*Vulpes vulpes*) population estimates and habitat requirements in several British cities. J. Anim. Ecol. 55, 575-591.

158. Abay, G. Y., Bauer, H., Gebrihiwot, K., & Deckers, J. (2011). Peri-urban spotted hyena (*Crocuta crocuta*) in northern Ethiopia: diet, economic impact, and abundance. European Journal of Wildlife Research, 57(4), 759-765.

159. Herr, J., Schley, L., Engel, E. & Roper, T.J. (2010). Den preferences and denning behaviour in urban stone martens (*Martes foina*). Mamm. Biol. 75, 138-145. Herr, J., Schley, L., Engel, E. & Roper, T.J. (2010).

160. Beckmann, J. P., & Berger, J. (2003). Rapid ecological and behavioural changes in carnivores: the responses of black bears (*Ursus* *americanus*) to altered food. Journal of Zoology, 261(2), 207-212.

161. Widdows, C. D., & Downs, C. T. (2015). A genet drive-through: are large spotted genets using urban areas for fast food? a dietary analysis. Urban ecosystems, 18(3), 907-920.

162.  Shanghai Agriculture and Forestry Bureau. 2004. Terrestrial wild plants and animals resources in Shanghai. Shanghai, China: Shanghai Scientific and Technology Press.

163. Cáceres, N. C., & Moura, M. O. (2003). Fruit removal of a wild tomato, *Solanum granulosoleprosum* Dunal (Solanaceae), by birds, bats and non-flying mammals in an urban Brazilian environment. Revista Brasileira de Zoologia, 20(3), 519-522.

164. Celuch, M., & Kanuch, P. (2005). Winter activity and roosts of the noctule (*Nyctalus noctula*) in an urban area (Central Slovakia). Lynx, n. s, 36, 39-45.

165. Coulson, G., Cripps, J. K., & Wilson, M. E. (2014). Hopping down the main street: eastern grey kangaroos at home in an urban matrix. Animals, 4(2), 272-291.

166. Duarte, M. H., & Young, R. J. (2011). Sleeping site selection by urban marmosets (*Callithrix penicillata*) under conditions of exceptionally high predator density. International Journal of Primatology, 32(2), 329-334.

167. Waite, T. A., Chhangani, A. K., Campbell, L. G., Rajpurohit, L. S., & Mohnot, S. M. (2007). Sanctuary in the city: urban monkeys buffered against catastrophic die-off during ENSO-related drought. EcoHealth, 4(3), 278-286.

168. Bueno, C., Faustino, M. T., & Freitas, S. (2013). Influence of landscape characteristics on capybara road-kill on highway BR-040, southeastern Brazil. Oecologia Australis, 17(2), 320-327.

169. Adkins, C.A. & Stott, P. (1998). Home ranges, movements and habitat associations of red foxes *Vulpes vulpes* in sub-urban Toronto. J. Zool., Lond. 244, 335-346.
